# Estimated 2023-2024 COVID-19 Vaccine Effectiveness in Adults

**DOI:** 10.1001/jamanetworkopen.2025.17402

**Published:** 2025-06-25

**Authors:** Ruth Link-Gelles, Elizabeth A. K. Rowley, Stephanie A. Irving, Nicola P. Klein, Shaun J. Grannis, Toan C. Ong, Sarah W. Ball, Malini B. DeSilva, Kristin Dascomb, Allison L. Naleway, Padma Koppolu, Ousseny Zerbo, Bruce Fireman, John Hansen, Julius Timbol, Lawrence Block, Brian E. Dixon, Thomas J. Duszynski, Katie S. Allen, David Mayer, Catia Chavez, Michelle Barron, Sarah E. Reese, Sean Chickery, Jonathan M. Davis, Allison Avrich Ciesla, Josephine Mak, Morgan Najdowski, Omobosola O. Akinsete, Charlene E. McEvoy, Inih J. Essien, Tamara Sheffield, Daniel Bride, Julie Arndorfer, Joshua Van Otterloo, Karthik Natarajan, Mark W. Tenforde, Jennifer DeCuir, Amanda B. Payne

**Affiliations:** 1 National Center for Immunizations and Respiratory Diseases, Centers for Disease Control and Prevention, Atlanta, Georgia; 2 Public Health Service Commissioned Corps, Rockville, Maryland; 3 Westat, Rockville, Maryland; 4 Kaiser Permanente Center for Health Research, Portland, Oregon; 5 Kaiser Permanente Vaccine Study Center, Kaiser Permanente Northern California Division of Research, Oakland; 6 Indiana University School of Medicine, Indianapolis; 7 Regenstrief Institute Center for Biomedical Informatics, Indianapolis, Indiana; 8 University of Colorado School of Medicine, Aurora; 9 HealthPartners Institute, Minneapolis, Minnesota; 10 Division of Infectious Diseases and Clinical Epidemiology, Intermountain Health, Salt Lake City, Utah; 11 Department of Health Policy and Management, Richard M Fairbanks School of Public Health, Indiana University, Indianapolis; 12 Department of Epidemiology, Richard M. Fairbanks School of Public Health, Indiana University, Indianapolis; 13 Eagle Health Analytics, San Antonio, Texas; 14 Department of Biomedical Informatics, Columbia University Irving Medical Center, New York, New York

## Abstract

**Importance:**

SARS-CoV-2 continues to evolve, population immunity changes, and COVID-19 vaccine formulas have been updated, necessitating ongoing COVID-19 vaccine effectiveness (VE) monitoring.

**Objectives:**

To evaluate the VE of 2023-2024 COVID-19 vaccines against COVID-19–associated emergency department (ED) and urgent care (UC) encounters, hospitalizations, and critical illness, including during XBB- and JN.1-predominant periods.

**Design, Setting, and Participants:**

This test-negative design VE case-control study was conducted using data from September 21, 2023, to August 22, 2024, from EDs, UC centers, and hospitals in 6 US health care systems. Eligible adults 18 years or older with COVID-19–like illness and molecular or antigen testing for SARS-CoV-2 were studied. Case patients were those with a positive molecular or antigen test result; control patients were those with a negative molecular test result.

**Exposure:**

Receipt of 2023-2024 (monovalent XBB.1.5) COVID-19 vaccination with products approved or authorized for use in the US.

**Main Outcomes and Measures:**

Main outcomes were COVID-19–associated ED and UC encounters, hospitalizations, and critical illness (admission to the intensive care unit or in-hospital death). VE was estimated comparing the odds of receipt of the 2023-2024 COVID-19 vaccine with no receipt among case and control patients.

**Results:**

Among 345 639 eligible ED and UC encounters in immunocompetent adults 18 years or older with COVID-19–like illness and available test results (median [IQR] age, 53 [34-71] years; 209 087 [60%] female), 37 096 (11%) had a positive SARS-CoV-2 test result. VE against COVID-19–associated ED and UC encounters was 24% (95% CI, 21%-26%) during 7 to 299 days after vaccination. Among 111 931 eligible hospitalizations in immunocompetent adults 18 years or older with COVID-19–like illness and available test results (median [IQR] age, 71 [58-81] years), 10 380 (9%) had a positive SARS-CoV-2 test result. During 7 to 299 days after vaccination, VE was 29% (95% CI, 25%-33%) against COVID-19–associated hospitalization and 48% (95% CI, 40%-55%) against COVID-19–associated critical illness. VE was highest 7 to 59 days after vaccination (VE against ED and UC encounters 49%; 95% CI, 46%-52%; hospitalization, 51%; 95% CI, 46%-56%; critical illness, 68%; 95% CI, 56%-76%) and then waned (VE 180-299 days after vaccination against ED and UC encounters, −7% [95% CI, −13% to −2%]; hospitalization, −4% [95% CI, −14% to 5%]; and critical illness, 16% [95% CI, −6 to 34%]).

**Conclusions and Relevance:**

In this case-control study of VE, 2023-2024 COVID-19 vaccines were estimated to provide additional effectiveness against medically attended COVID-19, with the highest and most sustained estimates against critical illness. These results highlight the importance of receiving recommended COVID-19 vaccination for adults 18 years or older.

## Key Points

**Question:**

What is the vaccine effectiveness (VE) of 2023-2024 COVID-19 vaccines against medically attended COVID-19, including during Omicron XBB and JN.1 sublineage predominance?

**Findings:**

This test-negative case-control study included 345 955 emergency department and urgent care encounters and 111 931 hospitalizations among adults with COVID-19–like illness. During 7 to 299 days after 2023-2024 COVID-19 vaccination, VE was 29% against COVID-19–associated emergency department and urgent care encounters, 30% against COVID-19–associated hospitalization, and 48% against COVID-19–associated critical illness, with VE being the highest 7 to 59 days after vaccination and waning against all outcomes.

**Meaning:**

In this study, the 2023-2024 COVID-19 vaccines were associated with fewer cases of medically attended COVID-19, with more robust outcomes for critical illness; however, VE waned over time, supporting recommendations for all adults to receive 2024 to 2025 COVID-19 vaccination.

## Teaser

This case-control study of US emergency departments, urgent care centers, and hospitals estimates vaccine effectiveness of the 2023-2024 COVID-19 vaccine and examines waning patterns and differences by age, immunocompromise status, and outcome to evaluate and inform vaccine recommendations.

## Introduction

In September 2023, the US Centers for Disease Control and Prevention’s (CDC’s) Advisory Committee on Immunization Practices (ACIP) recommended 2023-2024 COVID-19 vaccines for use in persons 6 months or older for prevention of COVID-19, including severe disease.^1^ Initially, a single dose was recommended for all persons 5 years or older without immunocompromising conditions, irrespective of previous COVID-19 vaccination history. Those with moderate or severe immunocompromise could receive additional doses. In February 2024, the ACIP updated their recommendations to include an additional dose of the 2023-2024 COVID-19 vaccine for persons 65 years or older.^2^ The 2023-2024 COVID-19 vaccine formulations, including messenger RNA (mRNA) vaccines (mRNA-1273 from Moderna and BNT162b2 from Pfizer-BioNTech) and a protein subunit vaccine (NVX-CoV2373 from Novavax), were monovalent and based on the SARS-CoV-2 Omicron variant XBB.1.5. XBB.1.5 predominated during the 2023 to 2024 respiratory season until spring 2024,^3^ when it was replaced by several descendants of XBB and Omicron BA.2.86, including JN.1 and KP.2, the lineages included in the 2024-2025 COVID-19 vaccine formulations.^4^ Given the continued evolution of SARS-CoV-2, monitoring COVID-19 vaccine effectiveness (VE) is important. Our objectives were to estimate the 2023-2024 COVID-19 VE and describe waning patterns and differences by age, immunocompromise status, and outcome to evaluate and inform vaccine recommendations.

## Methods

### Design, Setting, and Population

The Virtual SARS-CoV-2, Influenza, and Other Respiratory Viruses Network (VISION) is a multisite electronic health record (EHR)—based collaboration between the CDC and health care systems. This analysis used a test-negative design to estimate COVID-19 VE, including data from 6 health care systems in 8 states with a total of 373 emergency departments (EDs) and urgent care (UC) centers and 241 hospitals (eTable 1 in Supplement 1). Kaiser Permanente Northwest (Oregon and Washington), University of Colorado (Colorado), Intermountain Health (Utah), Regenstrief Institute (Indiana), HealthPartners (Minnesota and Wisconsin), and Kaiser Permanente Northern California (California) participated; most locations were private health care settings. Eligible encounters had one or more *International Statistical Classification of Diseases and Related Health Problems, Tenth Revision* (*ICD-10*) (eTable 2 in Supplement 1) discharge code related to COVID-19–like illness during September 21, 2023, to August 22, 2024. This study was reviewed by the CDC, deemed not human subjects research, and conducted consistent with applicable federal law and CDC policy.^5^ This study followed the Strengthening the Reporting of Observational Studies in Epidemiology (STROBE) reporting guideline.

Index date was defined as the earlier of (1) date of respiratory specimen collection with the most recent positive or negative SARS-CoV-2 test result within 10 days before to 72 hours after the encounter or (2) the encounter date. Cases were encounters with a positive SARS-CoV-2 molecular test result. Controls were encounters with a negative SARS-CoV-2 molecular test result. Variant-specific periods were defined by time when more than 50% of sequenced specimens nationally belonged to the respective lineage: XBB lineage predominance during September 21 to December 23, 2023, and JN.1 lineage predominance from December 24, 2023, to August 22, 2024.^6^

Eligible encounters were among persons 18 years or older at the index date. Repeat ED or UC visits within 7 days or repeat hospital admissions within 30 days from prior discharge were combined into a single encounter with the earliest index date. Patient characteristics, including demographics and underlying medical conditions, were obtained via EHR. Race and ethnicity categories included Black or African American, non-Hispanic; Hispanic or Latino, any race; White, non-Hispanic; other, non-Hispanic (American Indian or Alaska Native, Asian, Native Hawaiian or Other Pacific Islander, other, and multiple races); and unknown. Race and ethnicity were included in the study to assess and control for potential confounding and to assess generalizability of vaccine effectiveness findings across diverse populations. Patients were classified as immunocompromised based on *ICD-10* codes (eTable 3 in Supplement 1).

### Vaccination Status Classification

COVID-19 vaccination history was obtained via EHR, linked state or local immunization information systems, and, in some sites, claims data. Patients were considered vaccinated if they received a dose of a 2023-2024 COVID-19 vaccine approved or authorized in the US, including Moderna, Pfizer-BioNTech, and Novavax, 7 days or more before the index date. The referent group included encounters for persons who did not receive a 2023-2024 COVID-19 vaccine dose (including unvaccinated persons and those who received original monovalent or bivalent COVID-19 vaccines). Encounters in nonimmunocompromised adults 65 years or older were excluded if they received more than 1 dose of a 2023-2024 COVID-19 vaccine. For the main analysis, encounters in adults 65 years or older without immunocompromise were excluded if they received more than 2 doses of the 2023-2024 COVID-19 vaccine or received 2 doses of the 2023-2024 COVID-19 vaccines less than 120 days apart.

### Statistical Analysis

#### Main Analysis

Odds ratios were calculated using multivariable logistic regression models comparing the odds of 2023-2024 COVID-19 vaccination among cases and controls. VE was estimated as (1 − adjusted OR) × 100%. VE was estimated separately for COVID-19–associated ED or UC encounters and hospitalization, by immunocompromise status, and by XBB and JN.1 lineage predominance. VE against COVID-19–associated critical illness, defined as admission to an intensive care unit or in-hospital death, was calculated comparing odds of vaccination among cases with critical illness to hospitalized controls without critical illness. VE was stratified by time since vaccination (7-59 days, 60-119 days, and 120-299 days) and age at encounter (18-64 years and ≥65 years).

Standardized mean differences were calculated to assess meaningful differences in variable distributions by case and vaccination status; any variable with a standardized mean difference greater than 0.20 was considered for model inclusion. Final models were adjusted for age (as natural cubic splines), race and ethnicity (according to the EHR), sex, geographic region, and calendar day (as natural cubic splines).

#### Secondary Analysis of 2-Dose vs 1-Dose Relative VE

In a secondary analysis, we assessed the relative VE of a second dose of the same COVID-19 vaccine formulation in adults 65 years or older. To obtain sufficient statistical power, data from 2 respiratory virus seasons, 2022 to 2023 and 2023 to 2024, were combined. A second bivalent formula (original strain and Omicron BA.4/5) dose was recommended for adults 65 years or older under shared clinical decision-making (ie, as a “may receive” recommendation)^7^ from April 19 to September 13, 2023.^8^ A second 2023-2024 formula dose was recommended for all adults 65 years or older (ie, as a “should receive” recommendation) from March 1 to August 22, 2024.^9^ Previous analyses have shown that a first dose of bivalent COVID-19 vaccine had similar VE to a first dose of 2023-2024 COVID-19 vaccine.^10^

Encounters were included from adults 65 years or older who received 1 or 2 in-season COVID-19 doses 7 days or more before the index date. Persons were considered vaccinated with 1 or 2 in-season doses if they received 1 or 2 bivalent COVID-19 vaccine(s) and had an encounter during the 2022 to 2023 season or if they received 1 or 2 2023-2024 COVID-19 vaccine(s) and had an encounter during the 2023 to 2024 season. Encounters were excluded if they received no in-season COVID-19 vaccines, received 1 in-season COVID-19 vaccine less than 120 days prior (and thus were not yet eligible for a second dose), or received 2 doses of COVID-19 vaccine less than 120 days apart.^9^ Relative VE was calculated comparing receipt of 2 doses of the same in-season formulation (ie, 2 bivalent COVID-19 doses or 2 2023-2024 COVID-19 doses) with 1 dose of the same formula 120 days or more previously.

## Results

### Estimated Effectiveness of 2023-2024 COVID-19 Vaccines Against COVID-19–Associated ED and UC Encounters

Among 345 639 eligible ED and UC encounters (eFigure 1A in Supplement 1), median (IQR) patient age was 53 (34-71) years, 209 087 (60%) were female and 136 552 (40%) were male, 35 885 (10%) were Black or African American, 53 239 (15%) were Hispanic or Latino (any race), 210 395 (61%) were non-Hispanic White, 35 689 (10%) were non-Hispanic other race (American Indian or Alaska Native, Asian, Native Hawaiian or Other Pacific Islander, other, and multiple races), and 10 431 (3%) had unknown race (Table 1). December 2023 had the highest proportion of encounters (44 217 [13%]); most encounters (237 777 [69%]) occurred during JN.1 predominance. Of 37 096 ED and UC encounters (11%) with a positive SARS-CoV-2 test result, 5929 patients (16%) had received the 2023-2024 COVID-19 vaccine compared with 59 977 vaccinated control encounters (19%) with a negative SARS-CoV-2 test result. Most 2023-2024 COVID-19 doses received (65 750 [>99%]) were mRNA vaccines. Case patients were less likely to have at least 1 underlying medical condition (8304 cases [22%] vs 92 231 controls [30%]).

**Table 1.** Characteristics of ED and UC Encounters and Hospitalizations in Adults 18 Years or Older Without Documented Immunocompromise Included in the 2023-2024 COVID-19 Vaccine Effectiveness Analysis by Case and Vaccination Status, VISION Network, September 2023 to August 2024

Characteristic	SARS-CoV-2 status, No. (column %)			2023-2024 COVID-19 vaccination status, No. (row %)			Total, No. (column %)
	Cases (positive)	Controls (negative)	SMD	Did not receive 2023-2024 COVID-19 vaccination	Received 2023-2024 COVID-19 vaccination	SMD	
All ED and UC encounters	37 096	308 543	NA	279 733	65 906	NA	345 639
Age, median (IQR), y	55 (36-73)	53 (34-71)	0.080	48 (32-67)	71 (57-80)	0.858	53 (34-71)
Age group, y							
18-64	22 946 (62)	201 152 (65)	0.069	200 443 (89)	23 655 (11)	0.768	224 098 (65)
≥65	14 150 (38)	107 391 (35)		79 290 (65)	42 251 (35)		121 541 (35)
Sex							
Female	22 008 (59)	187 079 (61)	0.027	170 533 (82)	38 554 (18)	0.05	209 087 (60)
Male	15 088 (41)	121 464 (39)		109 200 (80)	27 352 (20)		136 552 (40)
Race and ethnicity							
Black or African American, non-Hispanic	3300 (9)	32 585 (11)	0.087	31 501 (88)	4384 (12)	0.262	35 885 (10)
Hispanic or Latino, any race	5335 (14)	47 904 (16)		45 576 (86)	7663 (14)		53 239 (15)
White, non-Hispanic	23 871 (64)	186 524 (60)		165 576 (79)	44 819 (21)		210 395 (61)
Other, non-Hispanic^a^	3651 (10)	32 038 (10)		27 724 (78)	7965 (22)		35 689 (10)
Unknown	939 (3)	9492 (3)		9356 (90)	1075 (10)		10 431 (3)
Site							
A	5274 (14)	35 911 (12)	0.418	30 628 (74)	10 557 (26)	0.512	41 185 (12)
B	10 009 (27)	44 276 (14)		47 726 (88)	6559 (12)		54 285 (16)
C	8429 (23)	111 811 (36)		89 861 (75)	30 379 (25)		120 240 (35)
D	2390 (6)	21 960 (7)		17 783 (73)	6567 (27)		24 350 (7)
E	5678 (15)	61 480 (20)		61 562 (92)	5596 (8)		67 158 (19)
F	5 316 (14)	33 105 (11)		32 173 (84)	6248 (16)		38 421 (11)
SVI of census tract of residence quartile^b^							
1	7518 (20)	58 791 (19)	0.208	49 221 (74)	17 088 (26)	0.321	66 309 (19)
2	6710 (18)	62 354 (20)		53 266 (77)	15 798 (23)		69 064 (20)
3	6080 (16)	61 032 (20)		54 103 (81)	13 009 (19)		67 112 (19)
4	4344 (12)	47 997 (16)		43 835 (84)	8506 (16)		52 341 (15)
Missing or unable to Geocode	12 444 (34)	78 369 (25)		79 308 (87)	11 505 (13)		90 813 (26)
2023-2024 COVID-19 vaccination status^c^							
Did not receive	31 167 (84)	248 566 (81)	0.091	279 733 (100)	0	NA	279 733 (81)
Received 7-299 d earlier	5929 (16)	59 977 (19)		0	65 906 (100)		65 906 (19)
Received 7-59 d earlier	1228 (3)	14 854 (5)		0	16 082 (100)		16 082 (5)
Received 60-119 d earlier	1521 (4)	16 132 (5)		0	17 653 (100)		17 653 (5)
Received 120-179 d earlier	875 (2)	12 940 (4)		0	13 815 (100)		13 815 (4)
Received 180-299 d earlier	2305 (6)	16 051 (5)		0	18 356 (100)		18 356 (5)
Vaccine type for 2023-2024 dose							
No. 2023-2024 dose	31 167 (84)	248 566 (81)	0.112	279 733 (100)	0	NA	279 733 (81)
mRNA-1273	1477 (25)	12 139 (20)		0	13 616 (100)		13 616 (21)
NVX-CoV2373	13 (<1)	143 (<1)		0	156 (100)		156 (<1)
BNT162b2	4439 (75)	47 695 (80)		0	52 134 (100)		52 134 (79)
SARS-CoV-2-positive	37 096 (100)	0	NA	31 167 (84)	5929 (16)	0.071	37 096 (11)
Month and year of encounter							
September 2023	1241 (3)	8833 (3)	0.489	10 062 (100)	12 (<1)	0.536	10 074 (3)
October 2023	3569 (10)	27 153 (9)		29 372 (96)	1350 (4)		30 722 (9)
November 2023	4569 (12)	30 558 (10)		30 618 (87)	4509 (13)		35 127 (10)
December 2023	6463 (17)	37 754 (12)		36 551 (83)	7666 (17)		44 217 (13)
January 2024	4846 (13)	36 642 (12)		33 603 (81)	7885 (19)		41 488 (12)
February 2024	2725 (7)	31 305 (10)		26 763 (79)	7267 (21)		34 030 (10)
March 2024	1545 (4)	30 802 (10)		24 994 (77)	7353 (23)		32 347 (9)
April 2024	989 (3)	26 574 (9)		20 761 (75)	6802 (25)		27 563 (8)
May 2024	1229 (3)	23 153 (8)		18 019 (74)	6363 (26)		24 382 (7)
June 2024	2339 (6)	19 580 (6)		15 855 (72)	6064 (28)		21 919 (6)
July 2024	4212 (11)	20 779 (7)		18 290 (73)	6701 (27)		24 991 (7)
August 2024	3369 (9)	15 406 (5)		14 841 (79)	3934 (21)		18 775 (5)
September 2024	0	4 (<1)		4 (100)	0		4 (<1)
SARS-CoV-2 lineage predominant period^d^							
XBB (September 21-December 23, 2023)	13 956 (38)	93 906 (30)	0.152	96 539 (90)	11 323 (10)	0.404	107 862 (31)
JN.1 (December 24, 2023-August 22, 2024)	23 140 (62)	214 637 (70)		183 194 (77)	54 583 (23)		237 777 (69)
No. of chronic medical condition categories^e^							
0	28 810 (78)	216 312 (70)	0.176	201 491 (82)	43 631 (18)	0.137	245 122 (71)
1	5694 (15)	64 925 (21)		55 543 (79)	15 076 (21)		70 619 (20)
2	1746 (5)	17 556 (6)		14 934 (77)	4368 (23)		19 302 (6)
3	562 (2)	6987 (2)		5596 (74)	1953 (26)		7549 (2)
≥4	284 (1)	2763 (1)		2169 (71)	878 (29)		3047 (1)
**All hospitalizations**							
Total No.	10 380	101 551	NA	87 718	24 213	NA	111 931
Age, median (IQR), y	76 (65-84)	71 (58-81)	0.294	69 (55-80)	77 (68-85)	0.541	71 (58-81)
Age group, y							
18-64	2463 (24)	37 089 (37)	0.282	35 303 (89)	4249 (11)	0.517	39 552 (35)
≥65	7917 (76)	64 462 (63)		52 415 (72)	19 964 (28)		72 379 (65)
Sex							
Female	5425 (52)	55 127 (54)	0.041	47 593 (79)	12 959 (21)	0.015	60 552 (54)
Male	4955 (48)	46 424 (46)		40 125 (78)	11 254 (22)		51 379 (46)
Race and ethnicity							
Black or African American, non-Hispanic	874 (8)	10 753 (11)	0.081	10 018 (86)	1609 (14)	0.212	11 627 (10)
Hispanic or Latino, any race	991 (10)	10 117 (10)		9097 (82)	2011 (18)		11 108 (10)
White, non-Hispanic	7369 (71)	69 997 (69)		59 628 (77)	17 738 (23)		77 366 (69)
Other, non-Hispanic^a^	970 (9)	8769 (9)		7173 (74)	2566 (26)		9739 (9)
Unknown	176 (2)	1915 (2)		1802 (86)	289 (14)		2091 (2)
Site							
A	518 (5)	4883 (5)	0.106	3572 (66)	1829 (34)	0.499	5401 (5)
B	1062 (10)	8306 (8)		7704 (82)	1664 (18)		9368 (8)
C	3560 (34)	32 781 (32)		25 487 (70)	10 854 (30)		36 341 (32)
D	428 (4)	5315 (5)		3959 (69)	1784 (31)		5743 (5)
E	3687 (36)	39 308 (39)		37 571 (87)	5424 (13)		42 995 (38)
F	1125 (11)	10 958 (11)		9425 (78)	2658 (22)		12 083 (11)
SVI of census tract of residence quartile^b^							17 978 (16)
1	1814 (17)	16 164 (16)	0.050	12 781 (71)	5197 (29)	0.289	20 860 (19)
2	1958 (19)	18 902 (19)		15 379 (74)	5481 (26)		20 785 (19)
3	1869 (18)	18 916 (19)		16 090 (77)	4695 (23)		15 878 (14)
4	1377 (13)	14 501 (14)		12 946 (82)	2932 (18)		
Missing or unable to Geocode	3362 (32)	33 068 (33)		30 522 (84)	5908 (16)		36 430 (33)
2023-2024 COVID-19 vaccination status^c^							
Did not receive	8480 (82)	79 238 (78)	0.092	87 718 (100)	0	NA	87 718 (78)
Received 7-299 d earlier	1900 (18)	22 313 (22)		0	24 213 (100)		24 213 (22)
Received 7-59 d earlier	417 (4)	5201 (5)	0.127	0	5618 (100)	0.159	5618 (5)
Received 60-119 d earlier	486 (5)	5745 (6)		0	6231 (100)		6231 (6)
Received 120-179 d earlier	306 (3)	4969 (5)		0	5275 (100)		5275 (5)
Received 180-299 d earlier	691 (7)	6398 (6)		0	7089 (100)		7089 (6)
Vaccine type for 2023-2024 dose							
No. 2023-2024 dose	8480 (82)	79 238 (78)	0.062	87 718 (100)	0	NA	87 718 (78)
mRNA-1273	420 (22)	5448 (24)		0	5868 (100)		5868 (24)
NVX-CoV2373	2 (<1)	48 (<1)		0	50 (100)		50 (<1)
BNT162b2	1478 (78)	16 817 (75)		0	18 295 (100)		18 295 (76)
SARS-CoV-2-positive	10 380 (100)	0	NA	8480 (82)	1900 (18)	0.064	10 380 (9)
Month and year of encounter							
September 2023	279 (3)	2808 (3)	0.450	3082 (100)	5 (<1)	0.553	3087 (3)
October 2023	1047 (10)	9151 (9)		9711 (95)	487 (5)		10 198 (9)
November 2023	1337 (13)	9587 (9)		9404 (86)	1520 (14)		10 924 (10)
December 2023	1787 (17)	10 939 (11)		10 306 (81)	2420 (19)		12 726 (11)
January 2024	1426 (14)	11 088 (11)		9745 (78)	2769 (22)		12 514 (11)
February 2024	852 (8)	9867 (10)		8126 (76)	2593 (24)		10 719 (10)
March 2024	535 (5)	10 191 (10)		7965 (74)	2761 (26)		10 726 (10)
April 2024	349 (3)	9413 (9)		7053 (72)	2709 (28)		9762 (9)
May 2024	366 (4)	8514 (8)		6308 (71)	2572 (29)		8880 (8)
June 2024	650 (6)	7346 (7)		5559 (70)	2437 (30)		7996 (7)
July 2024	960 (9)	7466 (7)		5933 (70)	2493 (30)		8426 (8)
August 2024	792 (8)	5181 (5)		4526 (76)	1447 (24)		5973 (5)
September 2024	0	0		0 (NA)	0 (NA)		0
SARS-CoV-2 lineage predominant period^d^							
XBB (September 21-December 23, 2023)	3967 (38)	29 781 (29)	0.189	29 977 (89)	3771 (11)	0.441	33 748 (30)
JN.1 (December 24, 2023-August 22, 2024)	6413 (62)	71 770 (71)		57 741 (74)	20 442 (26)		78 183 (70)
No. of chronic medical condition categories^e^							
0	1370 (13)	12 964 (13)	0.10	12 572 (88)	1762 (12)	0.338	14 334 (13)
1	1511 (15)	18 385 (18)		16 912 (85)	2984 (15)		19 896 (18)
2	2073 (20)	20 259 (20)		17 372 (78)	4960 (22)		22 332 (20)
3	2697 (26)	24 013 (24)		19 882 (74)	6828 (26)		26 710 (24)
≥4	2729 (26)	25 930 (26)		20 980 (73)	7679 (27)		28 659 (26)
Admitted to ICU	1487 (14)	19 240 (19)	0.124	16 681 (80)	4046 (20)	0.06	20 727 (19)
Receipt of invasive mechanical ventilatory support							
Yes	495 (5)	6541 (6)	0.105	5792 (82)	1244 (18)	0.375	7036 (6)
No	7202 (69)	65 862 (65)		54 127 (74)	18 937 (26)		73 064 (65)
Unknown	2683 (26)	29 148 (29)		27 799 (87)	4032 (13)		31 831 (28)
In-hospital death	501 (5)	4706 (5)	0.011	4216 (81)	991 (19)	0.035	5207 (5)

Abbreviations: ED, emergency department; ICU, intensive care unit; NA, not applicable; SMD, standardized mean difference; SVI, Social Vulnerability Index; UC, urgent care.

^a^ Other race includes American Indian or Alaska Native, Asian, Native Hawaiian or Other Pacific Islander, other, and multiple races.

^b^ The SVI is defined based on the census tract of residence. The Centers for Disease Control and Prevention and Agency for Toxic Substances and Disease Registry SVI uses 16 US Census variables to determine social vulnerability for each census tract. Higher SVI values correspond to higher social vulnerability, which refers to the potential negative effects on communities caused by external stresses on human health.

^c^ SMD for vaccination status by case or control status compares the following categories: no 2023 to 2024 COVID-19 vaccine dose, 2023 to 2024 dose 7-59 days earlier, 2023 to 2024 dose 60 to 119 days earlier, 2023 to 2024 dose 120 to 179 days earlier, and 2023 to 2024 dose 180 to 299 days earlier.

^d^ Variant-specific periods were defined by time when more than 50% of sequenced specimens nationally belonged to the respective lineage.

^e^ Underlying medical condition categories were cardiovascular, cerebrovascular, endocrine, gastrointestinal, hematologic, musculoskeletal, neurologic, pulmonary, and renal.

**Figure 1.**
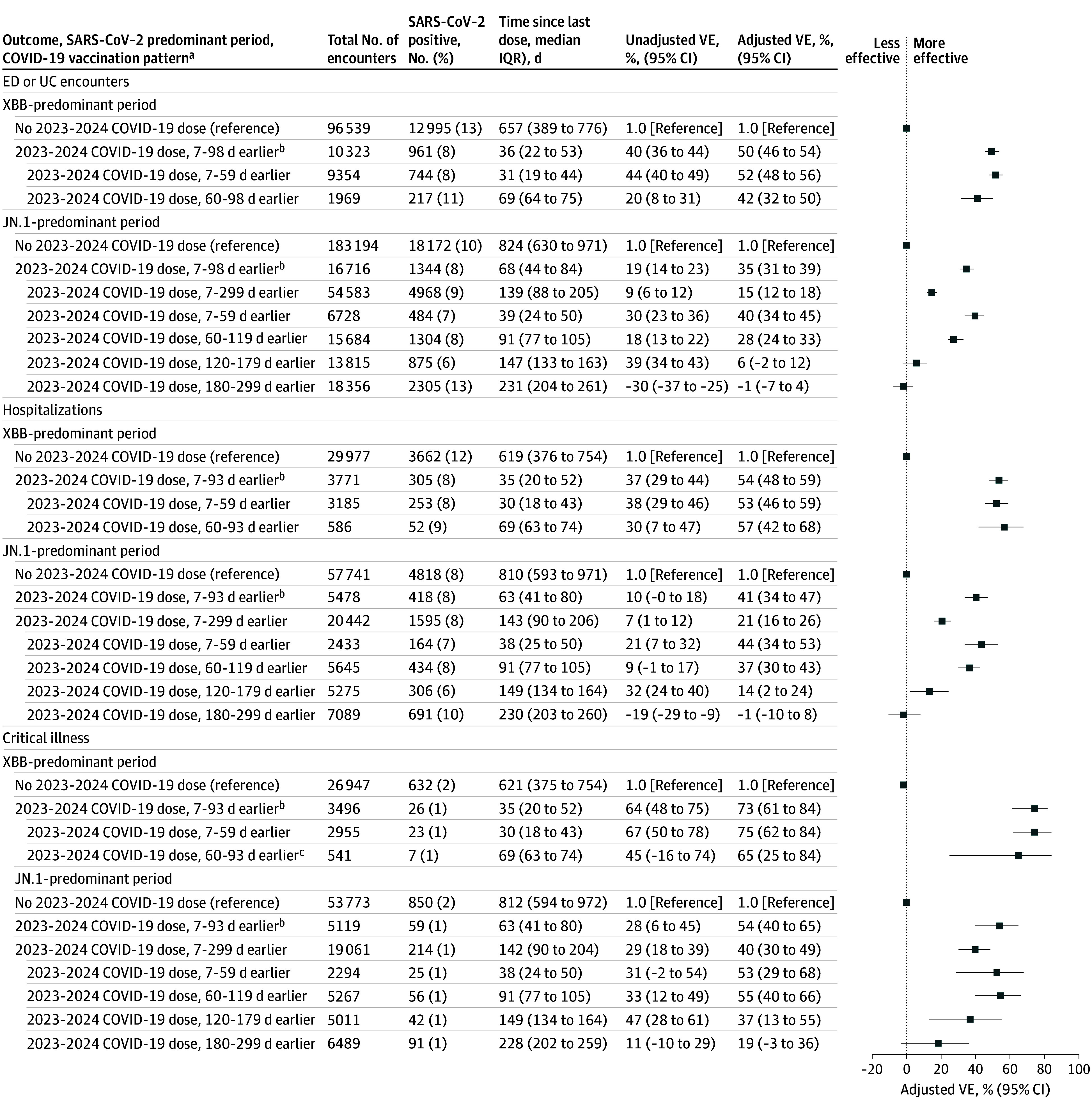
Vaccine Effectiveness (VE) by SARS-CoV-2 Variant Predominant Period Among Adults 18 Years or Older, September 2023 to August 2024 VE estimates were adjusted for age, race and ethnicity, sex, geographic region, calendar time. Error bars indicate 95% CIs. ED indicates emergency department; UC, urgent care. ^a^Variant-specific periods were defined by time when more than 50% of sequenced specimens nationally belonged to the respective lineage: September 21 to December 23, 2023 (XBB predominance) and December 24, 2023, to August 22, 2024 (JN.1 predominance) ^b^Due to the timing of the 2023-2024 COVID-19 vaccine recommendations and emergence of JN.1, VE during the XBB-predominant period could only be estimated to 98 days and 93 days past vaccination in the ED and UC as well as the hospital settings, respectively. To compare similar periods after dosing, we have included this abbreviated time period in the JN.1-predominant period estimates in addition to the full (ie, 7-299 day) period. ^c^Some estimates are imprecise, which might be due to a relatively small number of persons in each level of vaccination or case status. This imprecision indicates that the actual VE could be substantially different from the point estimate shown, and estimates should therefore be interpreted with caution. Additional data accrual could increase precision and allow more precise interpretation.

VE against COVID-19–associated ED and UC encounters was 24% (95% CI, 21%-26%) in the 7 to 299 days after vaccination and was similar in those aged 18 to 64 and 65 years or older (eFigure 2 in Supplement 1). During 7 to 59 days after vaccination, VE was 49% (95% CI, 45%-52%), decreasing to −7% (95% CI, −13% to −2%) 180 to 299 days after vaccination. During XBB predominance, VE against ED and UC encounters among adults 18 years or older was 50% (95% CI, 46%-54%) during 7 to 98 days after vaccination (Figure 1). Due to the timing of the XBB sublineage decrease and JN.1 emergence, VE during 99 to 299 days after vaccination could not be calculated during XBB predominance. During JN.1 predominance, VE against ED and UC encounters was 35% (95% CI, 31%-39%) during 7 to 98 days after vaccination, a comparable timeframe to that available during XBB predominance, and 15% (95% CI, 12%-18%) during the full 7 to 299 days after vaccination.

### Estimated Effectiveness of 2023-2024 COVID-19 Vaccines Against COVID-19–Associated Hospitalization

Among 111 931 eligible hospitalizations (eFigure 1B in Supplement 1), the median (IQR) patient age was 71 (58-81) years and 60 552 (54%) were female (Table 1). December 2023 and January 2024 had the most encounters (12 726 in December 2023 and 12 514 in January 2024 [11% each]); most encounters (78 183 [70%]) occurred during JN.1 predominance. Of 10 380 hospitalizations (9%) with a positive SARS-CoV-2 test result, 1900 (18%) had received the 2023-2024 COVID-19 vaccine compared with 22 313 control encounters (22%) with a negative SARS-CoV-2 test result. Most 2023-2024 COVID-19 doses received (24 163 [99%]) were mRNA vaccines. Controls were more likely to be admitted to the intensive care unit (1487 cases [14%] vs 19 240 controls [19%]) and had a similar likelihood of receiving invasive mechanical ventilatory support (495 cases [5%] and 6541 controls [6%]). There were 501 deaths among cases.

**Figure 2.**
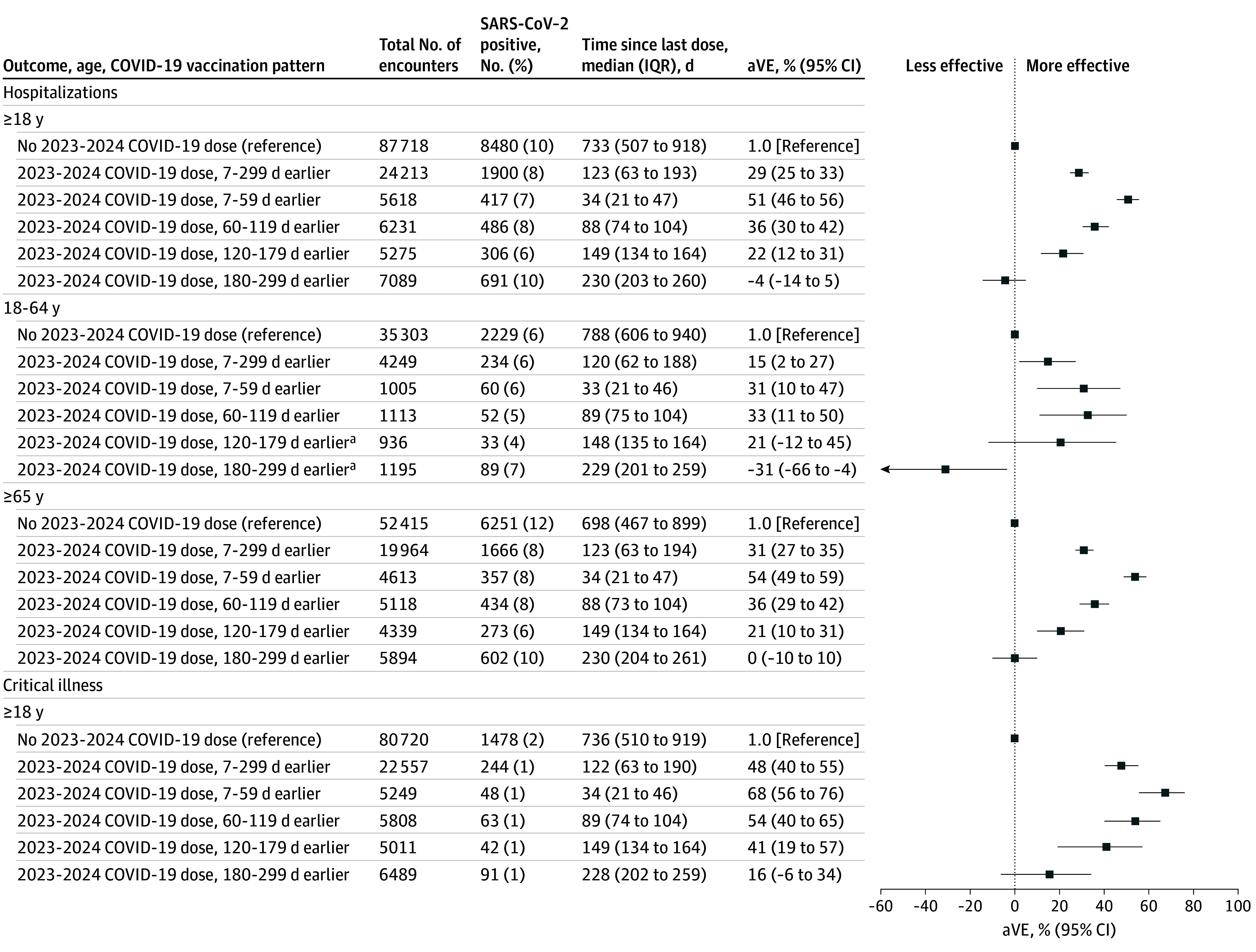
Vaccine Effectiveness Among Adults 18 Years or Older Without Documented Immunocompromise, September 2023- August 2024 Vaccine effectiveness estimates were adjusted for age, race and ethnicity, sex, geographic region, calendar time. Error bars indicate 95% CIs. aVE indicates adjusted vaccine effectiveness; ED, emergency department; UC, urgent care. ^a^Some estimates are imprecise, which might be due to a relatively small number of persons in each level of vaccination or case status. This imprecision indicates that the actual VE could be substantially different from the point estimate shown, and estimates should therefore be interpreted with caution. Additional data accrual could increase precision and allow more precise interpretation.

VE against COVID-19–associated hospitalizations was 29% (95% CI, 25%-33%) during 7 to 299 days after vaccination (Figure 2). During 7 to 59 days after vaccination, VE was 51% (95% CI, 46%-56%), decreasing to −4% (95% CI, −14 to 5%) 180 to 299 days after vaccination. Unlike the ED and UC setting, VE point estimates against hospitalization were generally higher among those 65 years or older vs those aged 18 to 64 years. At 7 to 59 days after vaccination, VE against hospitalization was 54% (95% CI, 49%-59%) for those 65 years or older vs 31% (95% CI, 10%-47%) for those aged 18 to 64 years. VE was similar across the age groups 60 to 119 days after a dose, with diminishing precision in younger adults at later time points since dose.

During XBB predominance, VE during 7 to 93 days after vaccination was 54% (95% CI, 48%-59%) against hospitalizations and 73% (95% CI, 61%-82%) against critical illness (Figure 1). VE during the 94 to 299 days after vaccination could not be calculated during XBB predominance. During JN.1 predominance, VE during 7 to 93 days after vaccination was 41% (95% CI, 34%-47%) against hospitalizations and 54% (95% CI, 40%-65%) against critical illness. VE against critical illness, regardless of variant predominance, was 48% (95% CI, 40%-55%) during 7 to 299 days after vaccination. VE was 68% (95% CI, 56%-76%) during 7 to 59 days after vaccination and 16% (95% CI, −6 to 34%) at 180 to 299 days after vaccination (Figure 2).

### Estimated Effectiveness of 2023-2024 COVID-19 Vaccines Against COVID-19–Associated Hospitalization Among Adults With Immunocompromise

A total of 33 524 eligible hospitalizations were in adults with a documented immunocompromising condition (eTable 4 in Supplement 1); of these, 2547 (8%) were cases and 30 977 (92%) were controls. Immunocompromised cases were more likely to have an intensive care unit admission compared with nonimmunocompromised cases (523 [21%] vs 1487 [14%]). Immunocompromised persons were more likely to have received a 2023-2024 COVID-19 vaccine dose than nonimmunocompromised persons (8915 [27%] vs 24 213 [22%]), and immunocompromised controls were slightly more likely to have received a 2023-2024 COVID-19 vaccine dose compared with immunocompromised cases (594 cases [23%] vs 8321 controls [27%]). Point estimates for VE against COVID-19–associated hospitalizations in immunocompromised people tended to be somewhat lower than for nonimmunocompromised people; however, CIs overlapped (eFigure 3 in Supplement 1).

### Estimated Relative VE of a Second In-Season Dose in the Combined Season Analysis

**Figure 3.**
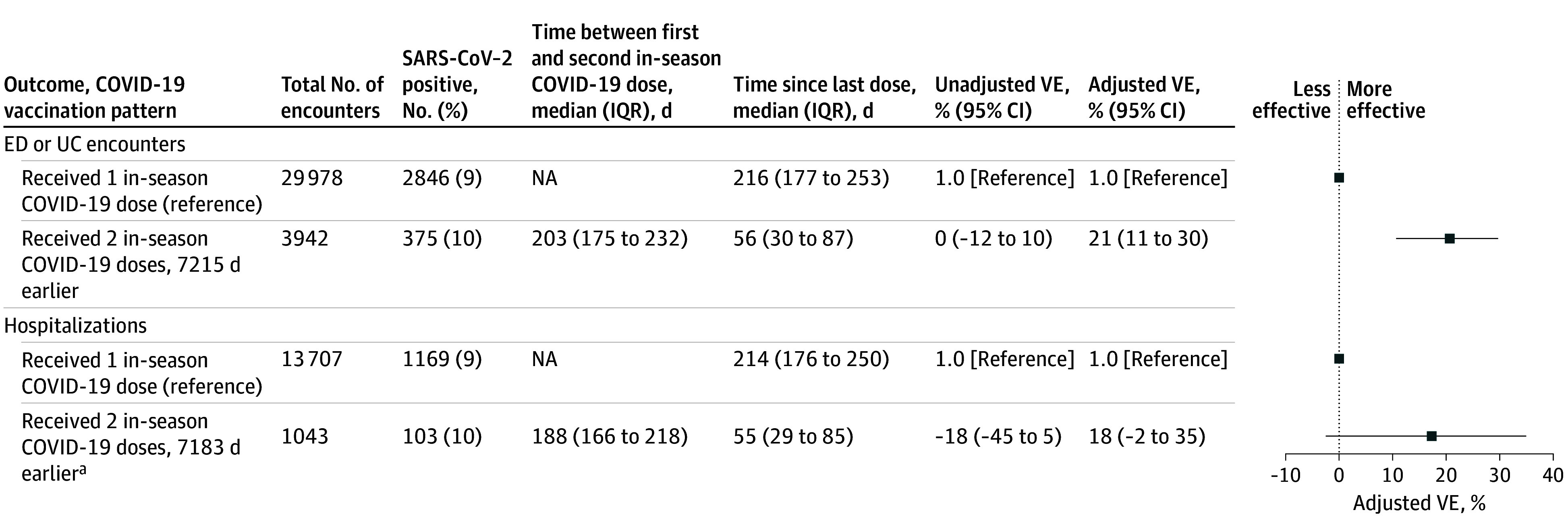
Relative 2- vs 1-Dose Vaccine Effectiveness (VE) Among Adults 65 Years or Older, April 2023 to August 2024 VE estimates were adjusted for age, race and ethnicity, sex, geographic region, calendar time. ED indicates emergency department; NA, not available; UC, urgent care.

Among 33 920 eligible ED and UC encounters in adults 65 years or older included in the combined season analysis (Table 2), 14 641 (43%) were from the bivalent period and 19 279 (57%) were from the 2023 to 2024 period (eFigure 4A in Supplement 1). Of 3221 total cases (9%), 29 978 cases and 30 699 controls, 2846 cases (88%) and 27 132 controls (88%) had received 1 in-season COVID-19 vaccine dose 120 days or more before their encounter (and thus eligible for a second in-season dose); 3942 cases and controls (12%) received 2 in-season COVID-19 vaccine doses. Among 14 750 eligible hospitalizations in adults 65 years or older included in the combined season analysis, 5787 (39%) were from the bivalent period and 8963 (60%) were from the 2023 to 2024 period (eFigure 4B in Supplement 1). Of 1272 total cases and 13 478 total controls, 1169 cases (92%) and 12 538 controls (93%) had received 1 in-season COVID-19 vaccine 120 days or more before their encounter; 103 cases (8%) received 2 in-season COVID-19 vaccines vs 940 controls (7%). Adjusted relative VE of 2 vs 1 dose of in-season COVID-19 vaccine was 21% (95% CI, 11%-30%) against COVID-19–associated ED and UC encounters and 18% (95% CI, −2 to 35%) against COVID-19–associated hospitalization (Figure 3).

**Table 2.** Characteristics of ED and UC Encounters and Hospitalizations in Adults 65 Years or Older Without Documented Immunocompromise Included in the Combined Bivalent and Second Dose 2023-2024 COVID-19 Vaccine Effectiveness Analysis by Case and Vaccination Status, VISION Network, April 2023 to August 2024

Characteristic	SARS-CoV-2 status, No. (column %)			COVID-19 vaccination status, No. (row %)			Total, No. (column %)
	Cases (positive)	Controls (negative)	SMD	Received 1 dose of in-season^a^ COVID-19 vaccine (bivalent or 2023-2024)	Received 2 doses of in-season^a^ COVID-19 vaccine (bivalent or 2023-2024)	SMD	
**All ED and UC encounters**							
Total No.	3221	30 699	NA	29 978	3942	NA	33 920
Age, median (IQR), y	78 (72-84)	78 (72-84)	0.052	78 (72-84)	78 (72-84)	0.021	78 (72-84)
Sex							
Female	1658 (51)	16 973 (55)	0.077	16 558 (89)	2073 (11)	0.053	18 631 (55)
Male	1563 (49)	13 726 (45)		13 420 (88)	1869 (12)		15 289 (45)
Race and ethnicity							
Black or African American, non-Hispanic	180 (6)	1823 (6)	0.025	1809 (90)	194 (10)	0.097	2003 (6)
Hispanic or Latino, any race	316 (10)	2896 (9)		2909 (91)	303 (9)		3212 (9)
White, non-Hispanic	2299 (71)	22 077 (72)		21 411 (88)	2965 (12)		24 376 (72)
Other, non-Hispanic^b^	399 (12)	3649 (12)		3601 (89)	447 (11)		4048 (12)
Unknown	27 (1)	254 (1)		248 (88)	33 (12)		281 (1)
Site							
A	525 (16)	4362 (14)	0.305	4292 (88)	595 (12)	0.356	4887 (14)
B	635 (20)	3090 (10)		3503 (94)	222 (6)		3725 (11)
C	1546 (48)	17 761 (58)		16 946 (88)	2361 (12)		19 307 (57)
D	358 (11)	3363 (11)		3057 (82)	664 (18)		3721 (11)
E	157 (5)	2123 (7)		2180 (96)	100 (4)		2280 (7)
F^c^	0	0		NA	NA		0
SVI of census tract of residence quartile^d^							
1	910 (28)	7235 (24)	0.120	7105 (87)	1040 (13)	0.121	8145 (24)
2	615 (19)	6006 (20)		5866 (89)	755 (11)		6621 (20)
3	470 (15)	4712 (15)		4653 (90)	529 (10)		5182 (15)
4	229 (7)	2770 (9)		2733 (91)	266 (9)		2999 (9)
Missing or unable to geocode	997 (31)	9976 (32)		9621 (88)	1352 (12)		10 973 (32)
COVID-19 vaccination status							
Received 1 bivalent dose ≥120 d earlier	1185 (37)	11 723 (38)	0.068	12 908 (100)	0	1.242	12 908 (38)
Received 2 bivalent doses, 7-299 d earlier	135 (4)	1598 (5)		0	1733 (100)		1733 (5)
Received 1 2023-2024 dose ≥120 d earlier	1661 (52)	15 409 (50)		17 070 (100)	0		17 070 (50)
Received 2 2023-2024 doses, 7-299 d earlier	240 (7)	1969 (6)		0	2209 (100)		2209 (7)
Vaccine type for most recent in-season dose corresponding to included encounter							
mRNA-1273	770 (24)	6804 (22)	0.041	7041 (93)	533 (7)	0.611	7574 (22)
NVX-CoV2373	2 (<1)	17 (<1)		18 (95)	1 (5)		19 (<1)
BNT162b2	2396 (74)	23 370 (76)		22 919 (89)	2847 (11)		25 766 (76)
Multiple manufacturers	53 (2)	508 (2)		0	561 (100)		561 (2)
SARS-CoV-2-positive	3221 (100)	0	NA	2846 (88)	375 (12)	0.001	3221 (9)
COVID-19 vaccine formulation period							
Bivalent (April 26, 2023-September 13, 2023)	1320 (41)	13 321 (43)	0.049	12 908 (88)	1733 (12)	0.018	14 641 (43)
2023-2024 (March 8, 2024-August 22, 2024)	1901 (59)	17 378 (57)		17 070 (89)	2209 (11)		19 279 (57)
No. of chronic medical condition categories							
0	2188 (68)	17 672 (58)	0.219	17 400 (88)	2460 (12)	0.091	19 860 (59)
1	620 (19)	8211 (27)		7891 (89)	940 (11)		8831 (26)
2	227 (7)	2671 (9)		2602 (90)	296 (10)		2898 (9)
3	105 (3)	1246 (4)		1202 (89)	149 (11)		1351 (4)
≥4	81 (3)	899 (3)		883 (90)	97 (10)		980 (3)
**All hospitalizations**							
Total No.	1272	13 478	NA	13 707	1043	NA	14 750
Age, median (IQR), y	81 (76-87)	79 (73-86)	0.198	80 (73-86)	81 (75-86)	0.107	80 (73-86)
Sex							
Female	599 (47)	7147 (53)	0.119	7217 (93)	529 (7)	0.039	7746 (53)
Male	673 (53)	6331 (47)		6490 (93)	514 (7)		7004 (47)
Race and ethnicity							
Black or African American, non-Hispanic	72 (6)	759 (6)	0.089	786 (95)	45 (5)	0.101	831 (6)
Hispanic or Latino, any race	98 (8)	1041 (8)		1072 (94)	67 (6)		1139 (8)
White, non-Hispanic	920 (72)	10 057 (75)		10 163 (93)	814 (7)		10 977 (74)
Other, non-Hispanic^b^	173 (14)	1476 (11)		1544 (94)	105 (6)		1649 (11)
Unknown	9 (1)	145 (1)		142 (92)	12 (8)		154 (1)
Site							
A	84 (7)	1188 (9)	0.258	1148 (90)	124 (10)	0.481	1272 (9)
B	131 (10)	1089 (8)		1158 (95)	62 (5)		1220 (8)
C	794 (62)	7155 (53)		7423 (93)	526 (7)		7949 (54)
D	90 (7)	1138 (8)		1012 (82)	216 (18)		1228 (8)
E	173 (14)	2908 (22)		2966 (96)	115 (4)		3081 (21)
F^c^	0	0		0 (NA)	0 (NA)		0
SVI of census tract of residence quartile^d^							
1	270 (21)	2772 (21)	0.064	2716 (89)	326 (11)	0.58	3042 (21)
2	264 (21)	2583 (19)		2574 (90)	273 (10)		2847 (19)
3	181 (14)	2170 (16)		2147 (91)	204 (9)		2351 (16)
4	112 (9)	1250 (9)		1257 (92)	105 (8)		1362 (9)
Missing or unable to geocode	445 (35)	4703 (35)		5013 (97)	135 (3)		5148 (35)
COVID-19 vaccination status							
Received 1 bivalent dose ≥120 d earlier	456 (36)	5141 (38)	0.058	5597 (100)	0	1.654	5597 (38)
Received 2 bivalent doses 7-299 d earlier	18 (1)	172 (1)		0	190 (100)		190 (1)
Received 1 2023-2024 dose ≥120 d earlier	713 (56)	7397 (55)		8110 (100)	0		8110 (55)
Received 2 2023-2024 doses 7-299 d earlier	85 (7)	768 (6)		0	853 (100)		853 (6)
Vaccine type for most recent in-season dose corresponding to included encounter							
BNT162b2	279 (22)	3336 (25)	0.069	3509 (97)	106 (3)	0.656	3615 (25)
NVX-CoV2373	1 (<1)	18 (<1)		19 (100)	0		19 (<1)
BNT162b2	981 (77)	10 004 (74)		10 179 (93)	806 (7)		10 985 (74)
Multiple manufacturers	11 (1)	120 (1)		0	131 (100)		131 (1)
SARS-CoV-2 positive	1272 (100)	NA	NA	1169 (92)	103 (8)	0.047	1272 (9)
COVID-19 vaccine formulation period							
Bivalent (April 26, 2023-September 13, 2023)	474 (37)	5313 (40)	0.044	5597 (97)	190 (3)	0.512	5787 (39)
2023-2024 (March 8, 2024-August 22, 2024)	798 (63)	8165 (60)		8110 (90)	853 (10)		8927 (61)
No. of chronic medical condition categories							
0	54 (4)	779 (6)	0.179	799 (96)	34 (4)	0.137	833 (6)
1	77 (6)	1284 (10)		1267 (93)	94 (7)		1361 (9)
2	282 (22)	2360 (18)		2438 (92)	204 (8)		2642 (18)
3	377 (30)	3838 (28)		3894 (92)	321 (8)		4215 (29)
≥4	482 (38)	5217 (39)		5309 (93)	390 (7)		5699 (39)
Admitted to ICU	121 (10)	2302 (17)	0.224	2275 (94)	148 (6)	0.067	2423 (16)
Receipt of invasive mechanical ventilatory support							
Yes	24 (2)	591 (4)	0.241	580 (94)	35 (6)	0.269	615 (4)
No	1125 (88)	10 771 (80)		10 966 (92)	930 (8)		11 896 (81)
Unknown	123 (10)	2116 (16)		2161 (97)	78 (3)		2239 (15)
In-hospital death	48 (4)	686 (5)	0.065	688 (94)	46 (6)	0.031	734 (5)

Abbreviations: ED, emergency department; ICU, intensive care unit; NA, not applicable; SMD, standardized mean difference; SVI, Social Vulnerability Index; UC, urgent care.

^a^ Persons were considered vaccinated with 1 or 2 in-season doses if they received bivalent COVID-19 vaccine(s) during the 2022 to 2023 season and had an encounter during April 26 to September 13, 2023, or if they received 2023 to 2024 COVID-19 vaccine(s) during the 2023 to 2024 season and had an encounter during March 8 to August 22, 2024.

^b^ Other race includes American Indian or Alaska Native, Asian, Native Hawaiian or Other Pacific Islander, other, and multiple races.

^c^ One site had incomplete vaccination history data during 2022 to 2023 and was therefore excluded from the combined season analysis.

^d^ The SVI is defined based on the census tract of residence. The Centers for Disease Control and Prevention and Agency for Toxic Substances and Disease Registry SVI uses 16 US census variables to determine social vulnerability for each census tract. Higher SVI values correspond to higher social vulnerability, which refers to the potential negative effects on communities caused by external stresses on human health.

## Discussion

In this case-control study of medically attended COVID-19–like illness encounters from September 21, 2023, to August 22, 2024, vaccination with the 2023-2024 COVID-19 vaccines provided additional effectiveness against COVID-19–associated ED and UC encounters, hospitalizations, and critical illness among adults 18 years or older compared with no receipt of the 2023-2024 COVID-19 vaccine. As found with earlier formulations of COVID-19 vaccines, VE waned over time but appeared highest and most durable against critical illness.^11,12,13^ In a combined season analysis, a second bivalent or 2023 to 2024 dose in adults 65 years or older appeared to provide some additional effectiveness beyond a single dose.

From July to August 2023, just before the 2023-2024 COVID-19 vaccines were introduced, anti–SARS-CoV-2 antibody seroprevalence in adults in the US was high. In one analysis^14^ of a national blood donor cohort, antinucleocapsid antibody (ie, infection induced) seroprevalence was 89% for persons aged 16 to 49 years, 84% in persons aged 50 to 64 years, and 72% in persons 65 years or older. Thus, VE estimates should be interpreted as the additional benefit provided by the 2023-2024 COVID-19 vaccination above existing protection from prior vaccination and prior infection. Differences in seroprevalence between age groups may also explain the higher VE in those 65 years or older in some strata. If older adults have a lower prevalence of infection-induced antibodies and thus lower protection against COVID-19 than younger adults, they may benefit more from vaccination, resulting in a higher measured VE in older adults than younger adults. Differences in behaviors, for example, masking and social distancing, may also contribute to these differences in VE by age group.

Waning was apparent in this analysis, including some strata 179 to 299 days after vaccination in which point estimates were negative. VE can be measured in multiple ways but is essentially a comparison between rates or risk of disease in vaccinated vs unvaccinated (or less vaccinated) populations. Persons who did not receive 2023-2024 COVID-19 vaccines were more susceptible to SARS-CoV-2 infection soon after 2023-2024 COVID-19 vaccine rollout, which corresponds to the earlier time since dose strata, compared with those who received a 2023-2024 COVID-19 vaccine.^15^ Because prior SARS-CoV-2 infection provides some protection against future SARS-CoV-2 infection and COVID-19,^16,17^ higher infection rates in the comparator population (ie, those without 2023-2024 vaccines) early in the study period may have temporarily provided them with higher infection-induced protection and reduced the measured VE with greater time since dose, resulting in negative VE estimates further from vaccination. Results in this analysis, which did not control for prior infection, suggest that the risk of COVID-19 among people who are more than 6 months from their 2023 to 2024 vaccination appears to be higher than unvaccinated people who may have had more recent prior infection. However, controlling for prior SARS-CoV-2 infection generally increases measured VE,^17^ lending further evidence that the measured VE in this analysis is likely an underestimate of vaccination’s true protection. Lower VE in younger age groups, including negative VE, may be indicative of differences in who gets vaccinated or patterns of prior infection. Notably, none of the VE estimates against critical illness were negative, highlighting that updated COVID-19 vaccines continued to provide protection against the most severe outcomes and supporting COVID-19 vaccine recommendations.

The combined season analysis indicated some additional benefit of a second dose of the same formulation (bivalent or 2023-2024) in adults 65 years or older; however, the CIs were wide and crossed the null against hospitalization. The combined season analysis comparison group is adults who received a single in-season dose 120 days or more prior (based on the recommendation for a second in-season dose 4 months after the first dose)^8,9^; this relative VE is therefore the added benefit of a second in-season dose beyond remaining protection afforded by the first in-season dose (and from any other prior vaccine doses or infections). Notably, the referent group for the combined season analysis was approximately 215 days from their last dose compared with more than 600 days in the referent group for the main analysis. It is clear from bivalent and 2023 to 2024 season-specific analyses that some protection, especially for critical illness, remains from the first in-season dose beyond 120 days,^11,13^ which, along with differences in circulating variants during the later part of the seasons, may explain the low relative VE in the combined season analysis. Of note, during the 2024 to 2025 season, the ACIP recommended a second 2024-2025 COVID-19 vaccine dose 6 months after their last dose for those at highest risk of severe COVID-19: adults 65 years or older and people with moderate or severe immunocompromise.^18^

Multiple COVID-19 vaccines containing different seasonally predominant strain components have never been approved at the same time for the same population and indication in the US,^2^ precluding a direct VE comparison to determine the importance of variant and vaccine match.^19^ VE point estimates in this analysis were lower during JN.1 predominance vs XBB predominance period, even when stratifying by time since dose. VE was higher during both variant periods against critical illness but was lower during JN.1 vs XBB predominance. The 2023-2024 COVID-19 vaccines included a monovalent Omicron XBB.1.5 component,^1^ a closer genetic match to the XBB-related sublineages predominating during September 21 to December 23, 2023, than the JN.1-related sublineages predominating during December 24, 2023, to August 22, 2024. Taken together, this finding indicates that the XBB-containing vaccines may have provided more protection when predominating variants were genetically closer to the vaccine, although there may have been residual confounding in this analysis due to changes in recent prior infection patterns, circulating rates of disease during the different variant-predominant periods, differences in individuals who sought vaccination earlier or later in the season, longer time since vaccination during JN.1 vs XBB periods even within time strata, or testing practices.

### Limitations

This analysis had at least the following limitations. First, the analysis was based on EHR data, including *ICD-10* codes collected for clinical care and billing, which may have resulted in underascertainment or misclassification of underlying medical conditions or other important variables. Second, patient samples were not available for genomic testing; therefore, conclusions about VE during XBB and JN.1 were based on timing of predominance in national samples. However, our results align with previously published estimates based on patient samples.^17,20^ Third, although some confounding was controlled for in the models, residual confounding is possible, including by prior infection status, exposure risk (eg, crowding and masking), and other unmeasured confounders. Fourth, the combined season analysis assumes similar VE during the 2022 to 2023 and 2023 to 2024 seasons. Coverage of a second in-season dose was not high enough to estimate season-specific VE, and combining seasons may obscure differences; however, VE of a first dose of bivalent and the 2023-2024 COVID-19 vaccine doses was similar.^10^

## Conclusions

The 2023-2024 COVID-19 vaccines were associated with additional protection against COVID-19–associated ED and UC encounters, hospitalization, and critical illness beyond existing protection provided by prior vaccination or SARS-CoV-2 infection. Vaccines were associated with protection for immunocompromised and nonimmunocompromised adults and during XBB- and JN.1-predominant periods, although protection was lower during JN.1, emphasizing the likely importance of receiving updated COVID-19 vaccination.

## Notes

Supplement 1. **eTable 1.** Number of Emergency Departments, Urgent Cares, and Hospitals, by Participating VISION Study Site **eTable 2.** International Statistical Classification of Diseases and Related Health Problems, Tenth Revision (ICD-10) Used to Define COVID-19-Like Illness **eTable 3.** International Statistical Classification of Diseases and Related Health Problems, Tenth Revision (ICD-10) Used to Define **eTable 4.** Characteristics of Hospitalizations in Adults Aged ≥18 Years With Documented Immunocompromise Included in 2023-2024 COVID-19 Vaccine Effectiveness Analysis, by Case and Vaccination Status, VISION Network, September 2023–August 2024 **eFigure 1.** Flowchart for the Selection of Emergency Department/Urgent Care Encounters (A) and Hospitalizations (B) for Persons Aged ≥18 Years Included in 2023-2024 COVID-19 Vaccine Effectiveness Analysis **eFigure 2.** Vaccine Effectiveness Against Emergency Department and Urgent Care Encounters Among Adults Aged ≥18 Years Without Documented Immunocompromise, September 2023- August 2024 **eFigure 3.** Vaccine Effectiveness (VE) Against Laboratory-Confirmed COVID-19-Associated Hospitalization and Critical Illness, Among Adults Aged ≥18 Years With Documented Immunocompromise, September 21, 2023 to August 22, 2024 **eFigure 4.** Flowchart for the Selection of Emergency Department/Urgent Care Encounters (A) and Hospitalizations (B) Persons Aged ≥65 Years Included in the Combined Bivalent and 2023-2024 Second Dose COVID-19 Vaccine Effectiveness Analysis

Supplement 2.
Data Sharing Statement
