# Supplementary material for: Estimated 2023-2024 COVID-19 Vaccine Effectiveness in Adults
Source: JAMA Netw Open. 2025 Jun 25;8(6):e2517402. doi: 10.1001/jamanetworkopen.2025.17402 (PMC12199055; doi:10.1001/jamanetworkopen.2025.17402)
Supplement: Supplement 1. — eTable 1. Number of Emergency Departments, Urgent Cares, and Hospitals, by Participating VISION Study Site eTable 2. International Statistical Classification of Diseases and Related Health Problems, Tenth Revision (ICD-10) Used to Define COVID-19-Like Illness eTable 3. International Statistical Classification of Diseases and Related Health Problems, Tenth Revision (ICD-10) Used to Define eTable 4. Characteristics of Hospitalizations in Adults Aged ≥18 Years With Documented Immunocompromise Included in 2023-2024 COVID-19 Vaccine Effectiveness Analysis, by Case and Vaccination Status, VISION Network, September 2023–August 2024 eFigure 1. Flowchart for the Selection of Emergency Department/Urgent Care Encounters (A) and Hospitalizations (B) for Persons Aged ≥18 Years Included in 2023-2024 COVID-19 Vaccine Effectiveness Analysis eFigure 2. Vaccine Effectiveness Against Emergency Department and Urgent Care Encounters Among Adults Aged ≥18 Years Without Documented Immunocompromise, September 2023- August 2024 eFigure 3. Vaccine Effectiveness (VE) Against Laboratory-Confirmed COVID-19-Associated Hospitalization and Critical Illness, Among Adults Aged ≥18 Years With Documented Immunocompromise, September 21, 2023 to August 22, 2024 eFigure 4. Flowchart for the Selection of Emergency Department/Urgent Care Encounters (A) and Hospitalizations (B) Persons Aged ≥65 Years Included in the Combined Bivalent and 2023-2024 Second Dose COVID-19 Vaccine Effectiveness Analysis [file jamanetwopen-e2517402-s001.pdf]

Supplemental Online Content

Link-Gelles R, Rowley EAK, Irving SA, et al. Estimation of 2023 to 2024 COVID-19 vaccine effectiveness in adults. *JAMA Netw Open*. 2025;8(6):e2517402. doi:10.1001/jamanetworkopen.2025.17402

eTable 1. Number of Emergency Departments, Urgent Cares, and Hospitals, by Participating VISION Study Site

eTable 2. International Statistical Classification of Diseases and Related Health Problems, Tenth Revision (ICD-10) Used to Define COVID-19-Like Illness

eTable 3. International Statistical Classification of Diseases and Related Health Problems, Tenth Revision (ICD-10) Used to Define

eTable 4. Characteristics of Hospitalizations in Adults Aged ≥18 Years With Documented Immunocompromise Included in 2023-2024 COVID-19 Vaccine Effectiveness Analysis, by Case and Vaccination Status, VISION Network, September 2023–August 2024

eFigure 1. Flowchart for the Selection of Emergency Department/Urgent Care Encounters (A) and Hospitalizations (B) for Persons Aged ≥18 Years Included in 2023-2024 COVID-19 Vaccine Effectiveness Analysis

eFigure 2. Vaccine Effectiveness Against Emergency Department and Urgent Care Encounters Among Adults Aged ≥18 Years Without Documented Immunocompromise, September 2023-August 2024

eFigure 3. Vaccine Effectiveness (VE) Against Laboratory-Confirmed COVID-19-Associated Hospitalization and Critical Illness, Among Adults Aged ≥18 Years With Documented Immunocompromise, September 21, 2023 to August 22, 2024

eFigure 4. Flowchart for the Selection of Emergency Department/Urgent Care Encounters (A) and Hospitalizations (B) Persons Aged ≥65 Years Included in the Combined Bivalent and 2023-2024 Second Dose COVID-19 Vaccine Effectiveness Analysis

This supplemental material has been provided by the authors to give readers additional information about their work.

© 2025 Link-Gelles R et al. *JAMA Network Open*.

eTable 1. Number of Emergency Departments, Urgent Cares, and Hospitals, by Participating VISION Study Site

**VISION Site**	**Emergency departments/urgent cares, n (col %)**	**Hospitals, n (col %)**
**Site**		
A	29 (8)	8 (3)
B	49 (13)	22 (9)
C	23 (6)	23 (10)
D	153 (41)	71 (29)
E	97 (26)	105 (44)
F	22 (6)	12 (5)

© 2025 Link-Gelles R et al. *JAMA Network Open*.

eTable 2. International Statistical Classification of Diseases and Related Health Problems, Tenth Revision (ICD-10) Used to Define COVID-19-Like Illness

Description of diagnosis	ICD-10 codes
COVID-19 pneumonia	
Pneumonia due to SARS-associated coronavirus	J12.81
Pneumonia due to coronavirus disease 2019	J12.82
Influenza pneumonia	
Influenza due to identified novel influenza A virus with pneumonia	J09.X1
Influenza due to other identified influenza virus with pneumonia	J10.0*
Influenza due to unidentified influenza virus with pneumonia	J11.0*
Other viral pneumonia	J12.*
Pneumonia due to Streptococcus pneumoniae	J13
Pneumonia due to Hemophilus influenzae	J14
Bacterial pneumonia, not elsewhere classified	J15.*
Pneumonia due to other infectious organisms, not elsewhere classified	J16.*
Pneumonia in diseases classified elsewhere	J17
Pneumonia, unspecified organism	J18.*
Influenza disease	J09.*, J10.1, J10.2, J10.8*, J11.1, J11.2, J11.8*,
Acute respiratory distress syndrome	J80
Chronic obstructive pulmonary disease with acute exacerbation	J44.1
Asthma acute exacerbation	J45.21, J45.22, J45.31, J45.32, J45.41, J45.42, J45.51, J45.52, J45.901, J45.902
Respiratory failure	
Acute respiratory failure	J96.0*
Acute and chronic respiratory failure	J96.2*
Respiratory arrest	R09.2
Respiratory failure, unspecified	J96.9*
Other acute lower respiratory tract infections	
Respiratory syncytial virus	B97.4
Acute bronchitis	J20.*
Acute bronchiolitis	J21.*
Unspecified acute lower respiratory infection	J22
Bronchitis, not specified as acute or chronic	J40
COPD with acute lower respiratory infection	J44.0
Simple and mucopurulent chronic bronchitis	J41.*
Unspecified chronic bronchitis	J42
Emphysema	J43.*
Bronchiectasis	J47.*
Abscess of lung and mediastinum	J85.*
Pyothorax	J86.*
Acute and chronic sinusitis	J01.*, J32.*
Acute upper respiratory tract infections	J00.*, J02.*, J03.*, J04.*, J05*, J06.*

© 2025 Link-Gelles R et al. *JAMA Network Open*.

Acute respiratory illness signs and symptoms	
Hemoptysis	R04.2
Cough	R05, R05.1, R05.2, R05.4, R05.8, R05.9
Dyspnea unspecified	R06.00
Shortness of breath	R06.02
Acute respiratory distress	R06.03
Stridor	R06.1
Wheezing	R06.2
Other abnormalities of breathing	R06.8
Apnea, not elsewhere classified	R06.81
Tachypnea, NEC	R06.82
Other abnormalities of breathing/ Other symptoms involving head & neck	R06.89
Chest pain on breathing/ painful respiration	R07.1
Asphyxia and hypoxemia	R09.0*
Pleurisy	R09.1
Respiratory arrest	R09.2
Abnormal sputum	R09.3
Other specified symptoms and signs involving the circulatory and respiratory systems	R09.8*
Acute febrile illness signs and symptoms	
Fever	R50.*
Fever presenting with conditions classified elsewhere	R50.81
Fever unspecified	R50.9
Chills (w/o fever)	R68.83
Febrile convulsions	R56.0*
Acute non-respiratory signs and symptoms	
Diarrhea	R19.7
Disturbance of smell and taste	R43.*
Headache	R51.9
Myalgia	M79.10, M79.18
Other malaise	R53.81
Other fatigue	R53.83
Altered level of consciousness / altered mental status	R41.82, R40.0, R40.1
Weakness	R53.1
Nausea and Vomiting	R11.0, R11.10, R11.11, R11.15, R11.2
Rash and other nonspecific skin eruption	R21.*
Abdominal pain	R10.0, R10.1*, R10.2, R10.3*, R10.81*, R10.84, R10.9
Elevated C-reactive protein (CRP)	R79.82
Elevated sedimentation rate (ESR)	R70.0
Sepsis	R65.*
Shock, unspecified	R57.9

*Includes all sub-codes.

© 2025 Link-Gelles R et al. *JAMA Network Open*.

eTable 3. International Statistical Classification of Diseases and Related Health Problems, Tenth Revision (ICD-10) Used to Define Immunocompromise Status

**Description of diagnosis**	**ICD-10 codes**
Hematological malignancy	C81.*, C82.*, C83.*, C84.*, C85.*, C86.*, C88.*, C90.*, C91.*, C92.*, C93.*, C94.*, C95.*, C96.*, D46.*, D61.0*, D70.0, D61.2, D61.9, D71.*
Solid organ malignancy	C00.*, C01.*, C02.*, C03.*, C04.*, C05.*, C06.*, C07.*, C08.*, C09.*, C10.*, C11.*, C12.*, C13.*, C14.*, C15.*, C16.*, C17.*, C18.*, C19.*, C20.*, C21.*, C22.*, C23.*, C24.*, C25.*, C26.*, C27.*, C28.*, C29.*, C30.*, C31.*, C32.*, C33.*, C34.*, C35.*, C36.*, C37.*, C38.*, C39.*, C40.*, C41.*, C42.*, C43.*, C45.*, C46.*, C47.*, C48.*, C49.*, C50.*, C51.*, C52.*, C53.*, C54.*, C55.*, C56.*, C57.*, C58.*, C59.*, C60.*, C61.*, C62.*, C63.*, C64.*, C65.*, C66.*, C67.*, C68.*, C69.*, C70.*, C71.*, C72.*, C73.*, C74.*, C75.*, C76.*, C77.*, C78.*, C79.*, C7A.*, C7B.*, C80.*,Z51.0, Z51.1*, C4A.*
Transplant recipient	T86.0*, T86.1*, T86.2*, T86.3*, T86.4*, T86.5*, T86.81*, T86.85*, D47.Z1, Z48.2*, Z94.*, Z98.85
Rheumatologic/inflammatory disorder	D86.*, E85.1, E85.2, E85.3, E85.4, E85.8*, E85.9, G35.*, J67.9*, L40.54, L40.59, L93.0*, L93.2*, L94.*, M05.*, M06.*, M07.*, M08.*, M30.*, M31.3*, M31.5*, M32.*, M33.*, M34.*, M35.3*, M35.89, M35.9*, M46.0*, M46.1, M46.8*, M46.9*
HIV/AIDS	B20.*, B21.*, B22.*, B23.*, B24.*, B97.35, O98.7*, and Z21*
Other intrinsic immune condition or immunodeficiency	D27.9, D72.89, D80.*, D81.0, D81.1, D81.2, D81.4, D81.5, D81.6, D81.7, D81.8*, D81.9, D82.*, D83.*, D84.*, D87.89,D89.0, D89.1, D89.3, D89.4*, D89.8*, D89.9, K70.3*, K70.4*, K72.*, K74.3, K74.4, K74.5, K74.6*, N04.*, R18.0

*Includes all sub-codes.

© 2025 Link-Gelles R et al. *JAMA Network Open*.

eTable 4. Characteristics of Hospitalizations in Adults Aged ≥18 Years With Documented Immunocompromise Included in 2023-2024 COVID-19 Vaccine Effectiveness Analysis, by Case and Vaccination Status, VISION Network, September 2023–August 2024

Characteristic	SARS-CoV-2 status, No. (col %)			2023-2024 COVID-19 vaccination status, No. (row %)			Total, No. (col %)
	Cases (positive)	Controls (negative)	SMD	Did not receive 2023-2024 COVID-19 vaccination	Received 2023-2024 COVID-19 vaccination	SMD	
**All hospitalizations**	**2,547**	**30,977**		**24,609**	**8,915**		**33,524**
**Median age, years (IQR)**	73 (63, 81)	70 (60, 78)	0.198	68 (58, 77)	74 (66, 81)	0.483	70 (60, 79)
**Age group, y**							
18-64	715 (28)	10,872 (35)	0.152	9,755 (84)	1,832 (16)	0.426	11,587 (35)
≥65	1,832 (72)	20,105 (65)		14,854 (68)	7,083 (32)		21,937 (65)
**Female sex**	1,241 (49)	15,857 (51)	0.049	12,662 (74)	4,436 (26)	0.034	17,098 (51)
**Race and ethnicity**							
Black or African American, NH	212 (8)	2,900 (9)	0.081	2,470 (79)	642 (21)	0.148	3,112 (9)
Hispanic or Latino, any race	274 (11)	3,616 (12)		3,001 (77)	889 (23)		3,890 (12)
White, NH	1,803 (71)	20,945 (68)		16,411 (72)	6,337 (28)		22,748 (68)
Other, NH[a]	231 (9)	2,998 (10)		2,281 (71)	948 (29)		3,229 (10)
Unknown	27 (1)	518 (2)		446 (82)	99 (18)		545 (2)
**Site**							
A	142 (6)	1,875 (6)	0.103	1,283 (64)	734 (36)	0.438	2,017 (6)
B	329 (13)	3,057 (10)		2,702 (80)	684 (20)		3,386 (10)
C	984 (39)	12,599 (41)		9,083 (67)	4,500 (33)		13,583 (41)
D	143 (6)	1,850 (6)		1,306 (66)	687 (34)		1,993 (6)
E	571 (22)	7,229 (23)		6,674 (86)	1,126 (14)		7,800 (23)
F	378 (15)	4,367 (14)		3,561 (75)	1,184 (25)		4,745 (14)
**SVI of census tract of residence quartile[b]**							
1	467 (18)	5,941 (19)	0.049	4,208 (66)	2,200 (34)	0.268	6,408 (19)

© 2025 Link-Gelles R et al. *JAMA Network Open*.

2	517 (20)	6,146 (20)		4,633 (70)	2,030 (30)		6,663 (20)
3	457 (18)	5,862 (19)		4,640 (73)	1,679 (27)		6,319 (19)
4	334 (13)	4,218 (14)		3,498 (77)	1,054 (23)		4,552 (14)
Missing or Unable to Geocode	772 (30)	8,810 (28)		7,630 (80)	1,952 (20)		9,582 (29)
**2023-2024 COVID-19 vaccination status[c]**							
Did not receive	1,953 (77)	22,656 (73)	0.082	24,609 (100)	0 (0)	NA	24,609 (73)
Received, 7-299 days earlier	594 (23)	8,321 (27)		0 (0)	8,915 (100)		8,915 (27)
Received, 7-59 days earlier	135 (5)	1,848 (6)		0 (0)	1,983 (100)	0.183	1,983 (6)
Received, 60-119 days earlier	158 (6)	2,139 (7)		0 (0)	2,297 (100)		2,297 (7)
Received, 120-179 days earlier	107 (4)	1,844 (6)		0 (0)	1,951 (100)		1,951 (6)
Received, 180-299 days earlier	194 (8)	2,490 (8)		0 (0)	2,684 (100)		2,684 (8)
**Vaccine type for 2023-2024 dose**							
No 2023-2024 dose	1,953 (77)	22,656 (73)	0.094	24,609 (100)	0 (0)	10.072	24,609 (73)
Moderna	135 (5)	1,768 (6)		0 (0)	1,903 (100)		1,903 (6)
Novavax	0 (0)	14 (0)		0 (0)	14 (100)		14 (0)
Pfizer-BioNTech	455 (18)	6,478 (21)		0 (0)	6,933 (100)		6,933 (21)
Multiple manufacturers	4 (0)	61 (0)		0 (0)	65 (100)		65 (0)
**Count of 2023-2024 doses**							
0	1,953 (77)	22,656 (73)	0.082	24,609 (100)	0 (0)	3.141	24,609 (73)
1	566 (22)	7,956 (26)		0 (0)	8,522 (100)		8,522 (25)
2	28 (1)	365 (1)		0 (0)	393 (100)		393 (1)
**SARS-CoV-2-positive**	2,547 (100)	0 (0)	NA	1,953 (77)	594 (23)	0.049	2,547 (8)
**Month of encounter**							
September 2023	70 (3)	891 (3)	0.439	960 (100)	1 (0)	0.582	961 (3)
October 2023	249 (10)	2,905 (9)		2,992 (95)	162 (5)		3,154 (9)
November 2023	294 (12)	2,850 (9)		2,573 (82)	571 (18)		3,144 (9)
December 2023	406 (16)	3,141 (10)		2,672 (75)	875 (25)		3,547 (11)
January 2024	372 (15)	3,180 (10)		2,558 (72)	994 (28)		3,552 (11)
February 2024	180 (7)	2,911 (9)		2,174 (70)	917 (30)		3,091 (9)
March 2024	153 (6)	3,036 (10)		2,184 (68)	1,005 (32)		3,189 (10)
April 2024	95 (4)	2,854 (9)		1,960 (66)	989 (34)		2,949 (9)
May 2024	89 (3)	2,745 (9)		1,858 (66)	976 (34)		2,834 (8)

© 2025 Link-Gelles R et al. *JAMA Network Open*.

June 2024	168 (7)	2,350 (8)		1,614 (64)	904 (36)		2,518 (8)
July 2024	267 (10)	2,461 (8)		1,800 (66)	928 (34)		2,728 (8)
August 2024	203 (8)	1,653 (5)		1,263 (68)	593 (32)		1,856 (6)
September 2024							
**SARS-CoV-2 lineage predominant period[d]**							
XBB (September 21-December 23, 2023)	910 (36)	9,021 (29)	0.141	8,544 (86)	1,387 (14)	0.453	9,931 (30)
JN.1 (December 24, 2023-August 22, 2024)	1,637 (64)	21,956 (71)		16,065 (68)	7,528 (32)		23,593 (70)
**Number of chronic medical condition categories[e]**							
0	59 (2)	1,239 (4)	0.110	1,079 (83)	219 (17)	0.16	1,298 (4)
1	251 (10)	3,018 (10)		2,600 (80)	669 (20)		3,269 (10)
2	513 (20)	5,671 (18)		4,550 (74)	1,634 (26)		6,184 (18)
3	721 (28)	8,342 (27)		6,467 (71)	2,596 (29)		9,063 (27)
≥4	1,003 (39)	12,707 (41)		9,913 (72)	3,797 (28)		13,710 (41)
**Admitted to ICU**	523 (21)	7,461 (24)	0.085	6,055 (76)	1,929 (24)	0.07	7,984 (24)
**Receipt of invasive mechanical ventilation**							
Yes	223 (9)	3,139 (10)	0.054	2,673 (80)	689 (20)	0.315	3,362 (10)
No	1,977 (78)	23,396 (76)		17,829 (70)	7,544 (30)		25,373 (76)
Unknown	347 (14)	4,442 (14)		4,107 (86)	682 (14)		4,789 (14)
**In-hospital death**	254 (10)	3,579 (12)	0.052	2,951 (77)	882 (23)	0.068	3,833 (11)

Abbreviations: ED, emergency department; NA, not applicable; NH, non-Hispanic, ICU, intensive care unit; SMD, standardized mean difference; UC, urgent care.

[a] Other race includes American Indian or Alaska Native, Asian, Native Hawaiian or other Pacific Islander, other, and multiple races.

[b] The Social Vulnerability Index is defined based on the census tract of residence. The Centers for Disease Control and Prevention and Agency for Toxic Substances and Disease Registry Social Vulnerability Index uses 16 US census variables to determine social vulnerability for each census tract. Higher Social Vulnerability Index values correspond to higher social vulnerability, which refers to the potential negative effects on communities caused by external stresses on human health.

[c] SMD for vaccination status by case/control status compares the following categories: no 2023-2024 COVID-19 vaccine dose; 2023-2024 dose, 7-59 days earlier; 2023-2024 dose, 60-119 days earlier; 2023-2024 dose, 120-179 days earlier; 2023-2024 dose, 180-299 days earlier.

[d] Variant-specific periods were defined by time when >50% of sequenced specimens nationally belonged to the respective lineage.

[e] Underlying medical condition categories were cardiovascular, cerebrovascular, endocrine, gastrointestinal, hematological, musculoskeletal, neurological, pulmonary, renal.

© 2025 Link-Gelles R et al. *JAMA Network Open*.

eFigure 1. Flowchart for the Selection of Emergency Department/Urgent Care Encounters (A) and Hospitalizations (B) for Persons Aged ≥18 Years Included in 2023-2024 COVID-19 Vaccine Effectiveness Analysis

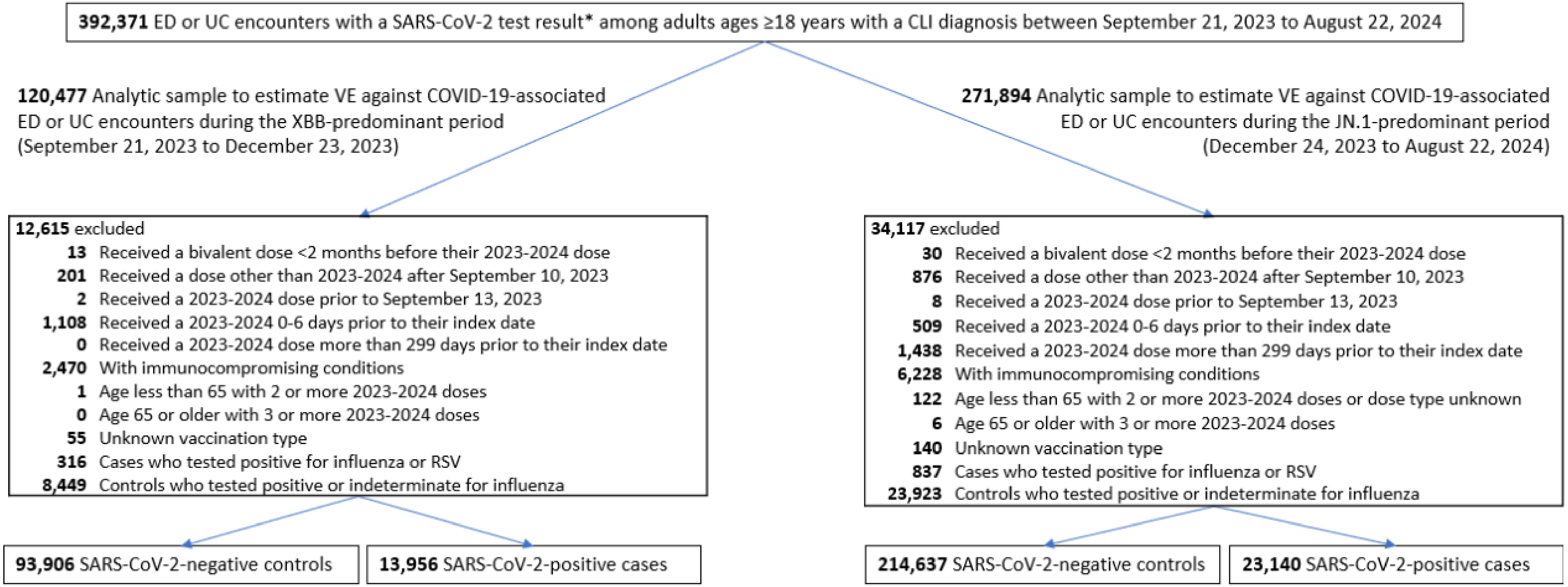

CLI indicates COVID-19-like-illness; ED, emergency department; UC, urgent care; VE, vaccine effectiveness.
* repeat ED or UC encounters collapsed within 7 days

© 2025 Link-Gelles R et al. *JAMA Network Open*.

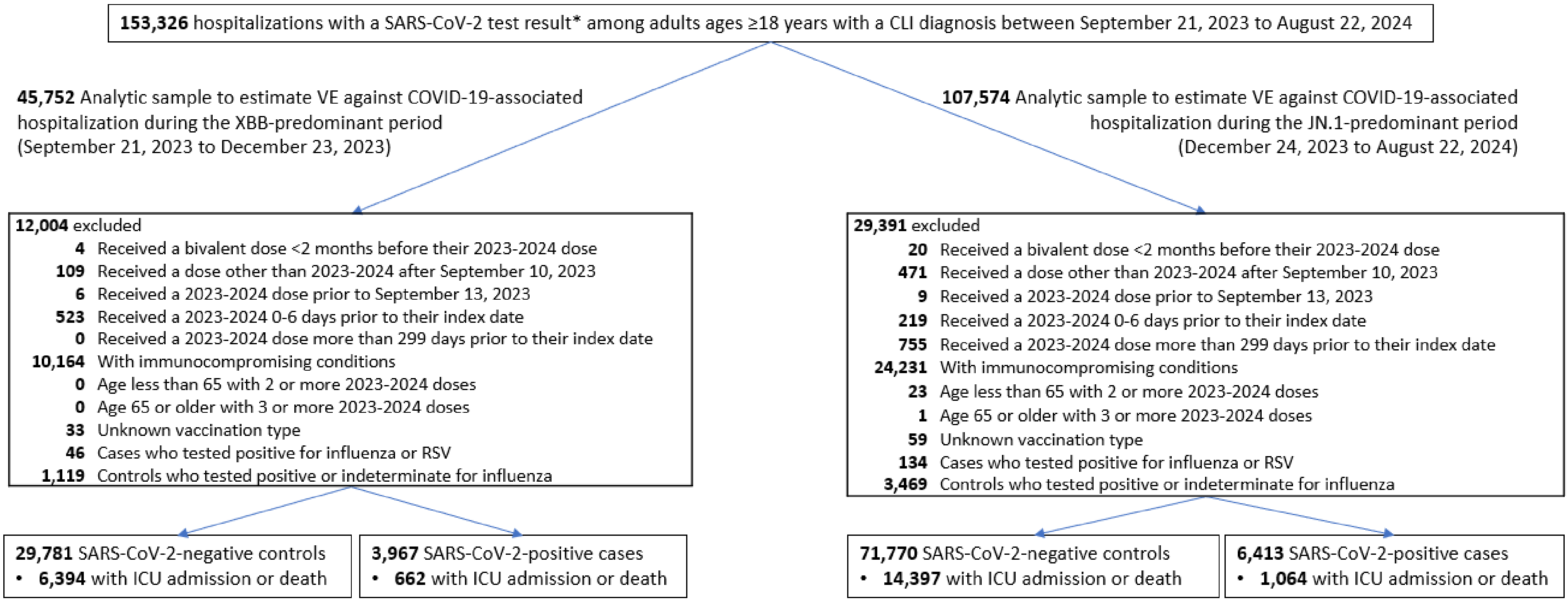

CLI indicates COVID-19-like-illness; ICU, intensive care unit; RSV, respiratory syncytial virus; VE, vaccine effectiveness

* repeat hospitalization collapsed within 30 days

© 2025 Link-Gelles R et al. *JAMA Network Open*.

eFigure 2. Vaccine Effectiveness Against Emergency Department and Urgent Care Encounters Among Adults Aged ≥18 Years Without Documented Immunocompromise, September 2023- August 2024

Outcome, age, COVID-19 vaccination pattern	Total encounters	SARS-CoV-2 positive, No. %	Median interval since last dose among vaccinated, days (IQR)	Adjusted VE, % (95% CI)
ED or UC encounters				
≥18y				
No 2023-2024 COVID-19 dose (ref)	279,733	31,167 (11)	756 (543-920)	ref
2023-2024 COVID-19 dose, 7-299 days earlier	65,906	5,929 (9)	117 (61-189)	24 (21 to 26)
2023-2024 COVID-19 dose, 7-59 days earlier	16,082	1,228 (8)	34 (21-47)	49 (46 to 52)
2023-2024 COVID-19 dose, 60-119 days earlier	17,653	1,521 (9)	88 (73-103)	26 (22 to 30)
2023-2024 COVID-19 dose, 120-179 days earlier	13,815	875 (6)	147 (133-163)	18 (12 to 24)
2023-2024 COVID-19 dose, 180-299 days earlier	18,356	2,305 (13)	231 (204-261)	-7 (-13 to -2)
18-64y				
No 2023-2024 COVID-19 dose (ref)	200,443	21,053 (10)	789 (624-934)	ref
2023-2024 COVID-19 dose, 7-299 days earlier	23,655	1,893 (8)	116 (59-187)	22 (18 to 26)
2023-2024 COVID-19 dose, 7-59 days earlier	5,952	360 (6)	34 (20-47)	55 (49 to 59)
2023-2024 COVID-19 dose, 60-119 days earlier	6,285	436 (7)	88 (74-104)	29 (22 to 36)
2023-2024 COVID-19 dose, 120-179 days earlier	4,971	256 (5)	147 (133-163)	22 (11 to 31)
2023-2024 COVID-19 dose, 180-299 days earlier	6,447	841 (13)	230 (203-260)	-24 (-34 to -14)
≥65y				
No 2023-2024 COVID-19 dose (ref)	79,290	10,114 (13)	669 (436-868)	ref
2023-2024 COVID-19 dose, 7-299 days earlier	42,251	4,036 (10)	117 (62-190)	25 (22 to 28)
2023-2024 COVID-19 dose, 7-59 days earlier	10,130	868 (9)	34 (21-47)	46 (41 to 50)
2023-2024 COVID-19 dose, 60-119 days earlier	11,368	1,085 (10)	88 (73-103)	25 (20 to 30)
2023-2024 COVID-19 dose, 120-179 days earlier	8,844	619 (7)	148 (133-163)	19 (11 to 26)
2023-2024 COVID-19 dose, 180-299 days earlier	11,909	1,464 (12)	232 (204-262)	2 (-5 to 8)

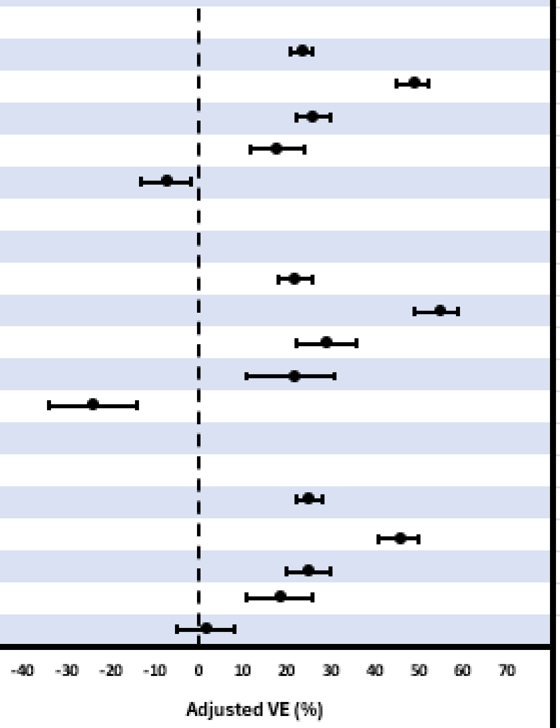

VE estimates were adjusted for age, race and ethnicity, sex, geographic region, calendar time. ED indicates emergency department; UC, urgent care.

© 2025 Link-Gelles R et al. *JAMA Network Open*.

eFigure 3. Vaccine Effectiveness (VE) Against Laboratory-Confirmed COVID-19-Associated Hospitalization and Critical Illness, Among Adults Aged ≥18 Years With Documented Immunocompromise, September 21, 2023 to August 22, 2024

Outcome, COVID-19 vaccination pattern	Total encounters	SARS-CoV-2 positive, No. %	Median interval since last dose among vaccinated, days (IQR)	Adjusted VE, % (95% CI)
**Hospitalizations**				
No 2023-2024 COVID-19 dose (ref)	24,609	1,953 (8)	702 (469-901)	ref
2023-2024 COVID-19 dose, 7-299 days earlier	8,915	594 (7)	125 (66-195)	20 (12 to 28)
2023-2024 COVID-19 dose, 7-59 days earlier	1,983	135 (7)	34 (21-46)	39 (26 to 49)
2023-2024 COVID-19 dose, 60-119 days earlier	2,297	158 (7)	89 (74-105)	25 (11 to 37)
2023-2024 COVID-19 dose, 120-179 days earlier	1,951	107 (5)	149 (134-164)	4 (-19 to 22)
2023-2024 COVID-19 dose, 180-299 days earlier	2,684	194 (7)	231 (203-261)	2 (-16 to 18)
**Critical illness**				
No 2023-2024 COVID-19 dose (ref)	23,163	507 (2)	703 (472-902)	ref
2023-2024 COVID-19 dose, 7-299 days earlier	8,441	120 (1)	125 (66-195)	40 (26 to 51)
2023-2024 COVID-19 dose, 7-59 days earlier[a]	1,876	28 (1)	34 (21-46)	54 (32 to 69)
2023-2024 COVID-19 dose, 60-119 days earlier[a]	2165	26 (1)	89 (74-105)	55 (32 to 70)
2023-2024 COVID-19 dose, 120-179 days earlier	1866	22 (1)	149 (134-164)	25 (-18 to 52)
2023-2024 COVID-19 dose, 180-299 days earlier	2534	44 (2)	230 (203-260)	11 (-25 to 37)

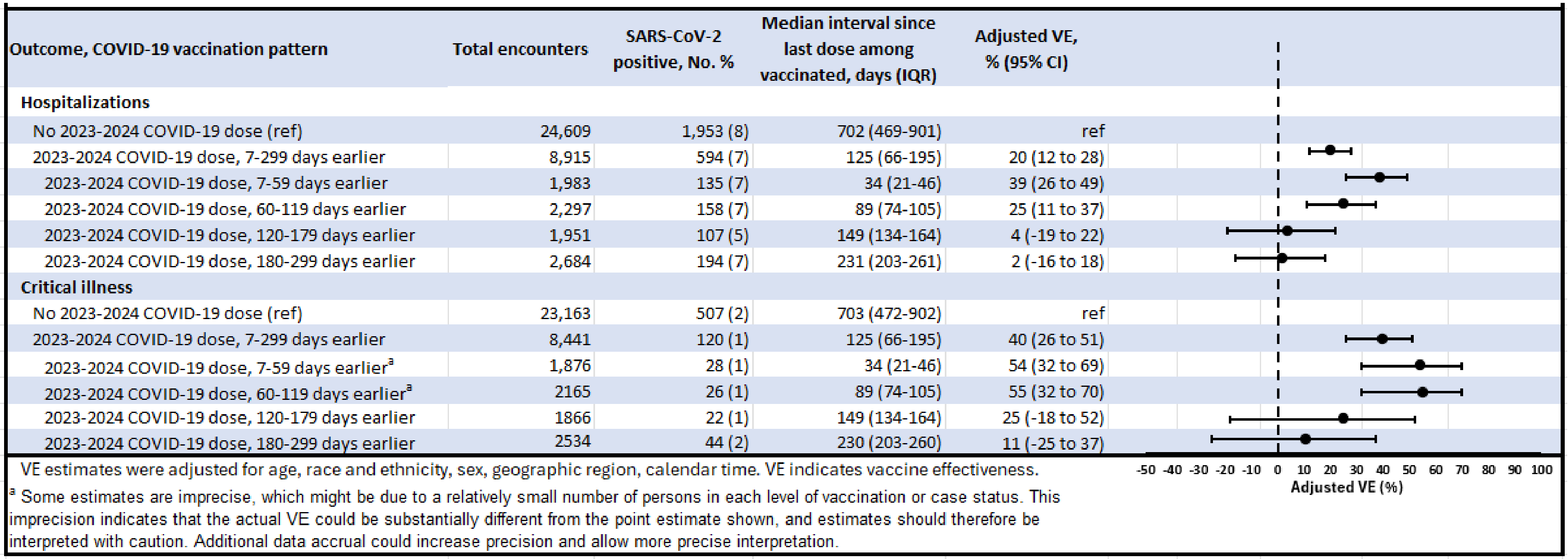

VE estimates were adjusted for age, race and ethnicity, sex, geographic region, calendar time. VE indicates vaccine effectiveness.

[a] Some estimates are imprecise, which might be due to a relatively small number of persons in each level of vaccination or case status. This imprecision indicates that the actual VE could be substantially different from the point estimate shown, and estimates should therefore be interpreted with caution. Additional data accrual could increase precision and allow more precise interpretation.

© 2025 Link-Gelles R et al. *JAMA Network Open*.

eFigure 4. Flowchart for the Selection of Emergency Department/Urgent Care Encounters (A) and Hospitalizations (B) Persons Aged ≥65 Years Included in the Combined Bivalent and 2023-2024 Second Dose COVID-19 Vaccine Effectiveness Analysis

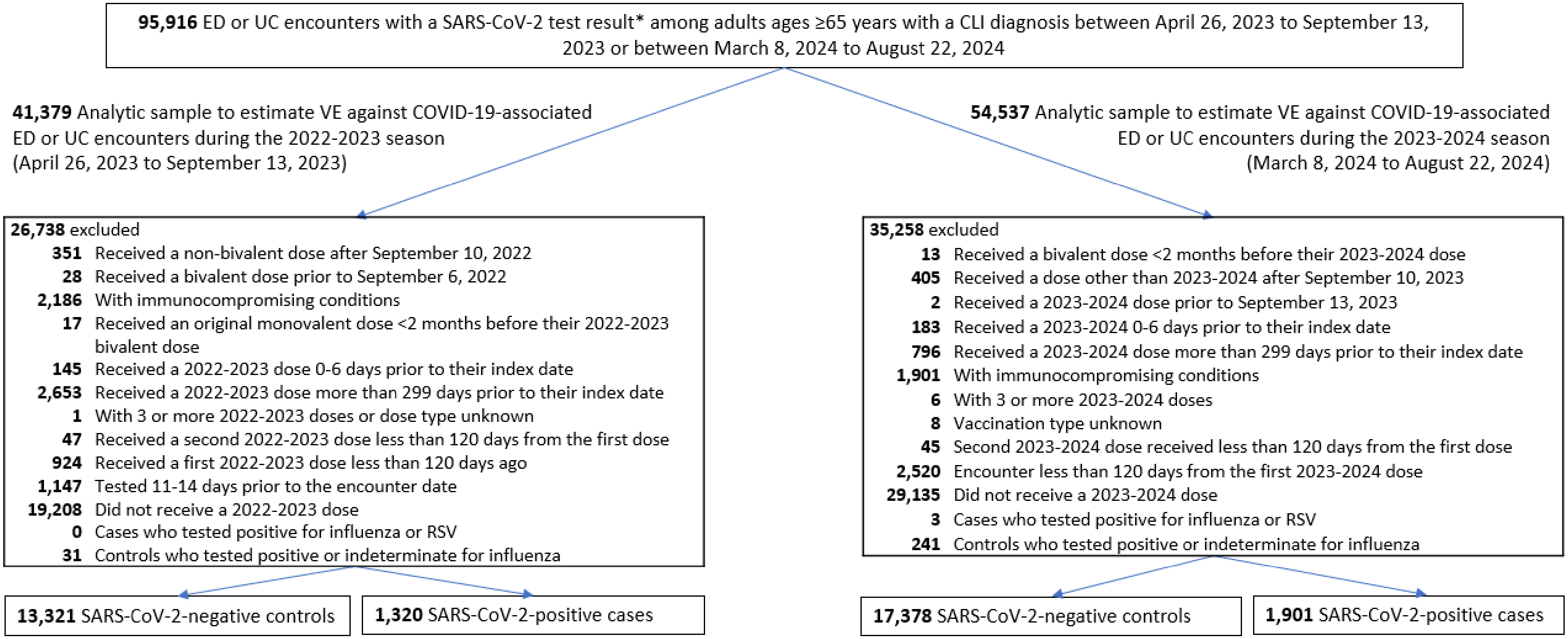

CLI indicates COVID-19-like-illness; ED, emergency department; RSV, respiratory syncytial virus; UC, urgent care; VE, vaccine effectiveness

* repeat ED or UC encounters collapsed within 7 days

© 2025 Link-Gelles R et al. *JAMA Network Open*.

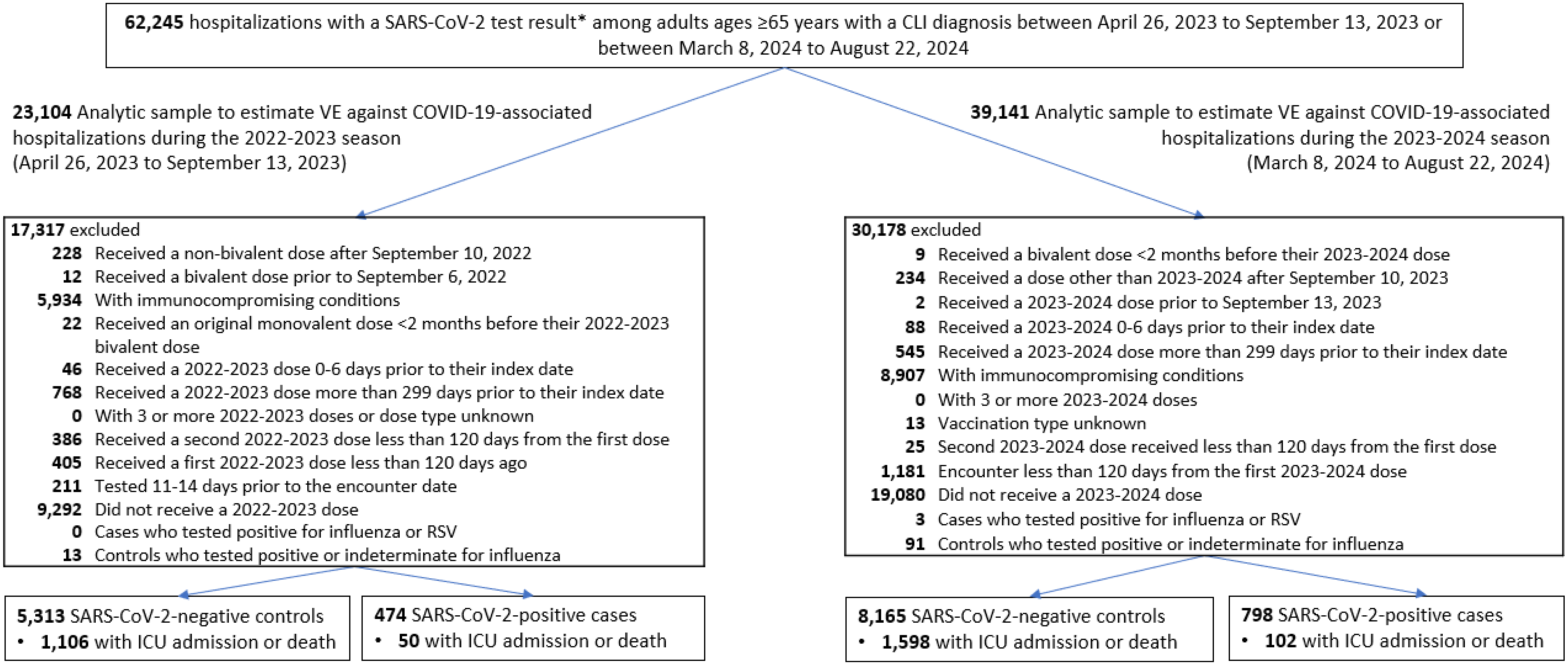

CLI indicates COVID-19-like-illness; ICU, intensive care unit; RSV, respiratory syncytial virus; VE, vaccine effectiveness
* repeat hospitalizations collapsed within 30 days

© 2025 Link-Gelles R et al. *JAMA Network Open*.
