# Supplementary material for: Estimated 2023-2024 COVID-19 Vaccine Effectiveness in Adults
Source: JAMA Netw Open. 2025 Jun 25;8(6):e2517402. doi: 10.1001/jamanetworkopen.2025.17402 (PMC12199055; doi:10.1001/jamanetworkopen.2025.17402)
Supplement: Supplement 2. — Data Sharing Statement [file jamanetwopen-e2517402-s002.pdf]

Data Sharing Statement

Data
Data available: No

Additional Information
Explanation for why data not available: Data collected for the study are not available. Data sharing agreements between the CDC and VISION partner institutions prohibit the CDC from making this dataset publicly available.
